# An exceptionally preserved 110 million years old praying mantis provides new insights into the predatory behaviour of early mantodeans

**DOI:** 10.7717/peerj.3605

**Published:** 2017-07-24

**Authors:** Marie K. Hörnig, Joachim T. Haug, Carolin Haug

**Affiliations:** 1 Zoological Institute and Museum, Cytology and Evolutionary Biology, Ernst-Moritz-Arndt Universität Greifswald, Greifswald, Germany; 2 Biocenter, Department of Biology II and GeoBio-Center, Ludwig-Maximilians-Universität München, Munich, Germany

**Keywords:** Mantodea, Dictyoptera sensu stricto, Raptorial appendage, Dictyoptera sensu lato, Fossil behaviour

## Abstract

Mantodeans or praying mantises are flying insects and well known for their raptorial behaviour, mainly performed by their first pair of thoracic appendages. We describe here a new, exceptionally preserved specimen of the early mantodean *Santanmantis axelrodi* Grimaldi, 2003 from the famous 110 million years old Crato Formation, Brazil. The incomplete specimen preserves important morphological details, which were not known in this specific form before for this species or any other representative of Mantodea. Unlike in modern representatives or other fossil forms of Mantodea not only the first pair of thoracic appendages shows adaptations for predation. The femora of the second pair of thoracic appendages bear numerous strong, erect spines which appear to have a sharp tip, with this strongly resembling the spines of the first pair of thoracic appendages. This indicates that individuals of *S. axelrodi* likely used at least two pairs of thoracic appendages to catch prey. This demonstrates that the prey-catching behaviour was more diverse in early forms of praying mantises than anticipated.

## Introduction

Praying mantises, or mantodeans, are iconic “flying insects” (Pterygota). They are especially famous for their evolutionary adaptations to predatory behaviour (Svenson & Whiting, 2004). Most apparent: their first pair of the three pairs of thoracic appendages is specialised for catching prey, each appendage being foldable and armed with massive spines.

Mantodea is an ingroup of Dictyoptera sensu stricto (sensu Béthoux, Klass & Schneider, 2009) and sister group to Blattodea (in a simplified neontological view), the group of modern cockroaches and termites (Lo et al., 2003; Lo et al., 2007; Grimaldi & Engel, 2005; Klass & Meier, 2006; Inward, Beccaloni & Eggleton, 2007; Djernæs et al., 2012; Djernæs, Klass & Eggleton, 2015; Legendre et al., 2015). Early representatives of Dictyoptera sensu lato (MK Hörnig, C Haug, JW Schneider & JT Haug, 2017, unpublished data) known from down to 315 million years ago strongly resemble modern cockroaches in many aspects (Rasnitsyn & Quicke, 2002; Grimaldi & Engel, 2005; Zhang, Schneider & Hong, 2013; Haug, Haug & Garwood, 2016; MK Hörnig, C Haug, JW Schneider & JT Haug, 2017, unpublished data), although the phylogenetic relationships of the Palaeozoic forms, the so-called ‘roachoids,’ are not resolved up to now (Grimaldi & Engel, 2005; Legendre et al., 2015).

In a simplified way we could say that mantises are nothing less than highly specialised cockroaches. The evolutionary transformation from a cockroach-like habitus to the highly specialised morphology of praying mantises most likely occurred via a step-wise character acquisition, not in a single event (Hörnig, Haug & Haug, 2013). The order in which these small steps occurred is luckily partly reconstructable via exceptionally preserved fossils of early mantodeans and dictyopterans branching off the evolutionary lineage towards Mantodea (e.g., Grimaldi, 2003; Grimaldi & Engel, 2005; Dittmann et al., 2015; Vršanský & Bechly, 2015; Bai et al., 2016; Delclòs et al., 2016).

The earliest presumed praying mantis remains have been described from the Jurassic (*Juramantis initialis* Vršanský, 2002 from Vršanský, 2002 and Vršanský, 2005; controversial, as only based on a fragmentary wing, see Vršanský, 2002 vs. Grimaldi, 2003). The Cretaceous period seems most important for understanding the early evolution of mantodeans (e.g., Grimaldi, 2003).

*Raptoblatta waddingtonae* Dittmann et al., 2015 is a rather roach-like species only known from the holotype, found in the Crato Formation, Brazil (Dittmann et al., 2015). Despite the overall cockroach-like habitus, the first pair of thoracopods strongly resembles that of mantodeans concerning shape of the femur, relative length of the tibia, and armature with spines on both leg elements. These similarities were interpreted as synapomorphies of *R*. *waddingtonae* and Mantodea by Dittmann et al. (2015). Lee (2016) on the contrary suggested that *R*. *waddingtonae* is an ingroup of Mesoblattinidae. Yet, Mesoblattinidae seems to be a kind of waste basket group (e.g., Grimaldi & Engel, 2005). Furthermore, Lee (2016) did not provide any explanations why the complex similarities shared between Mantodea and *R*. *waddingtonae* should be considered as convergencies. We therefore think that the synapomorphies suggested by Dittmann et al. (2015) are valid and consequently interpret *R*. *waddingtonae* as sistergroup to Mantodea. The tibiae of the first pair of thoracic appendages of *R*. *waddingtonae* only differ from those of mantodeans by lacking a prominent, curved distal spine, which therefore most likely represents an (aut-)apomorphy of Mantodea. Given this morphology, *R*. *waddingtonae* gives an important signal for the order of character acquisition along the evolutionary lineage towards Mantodea.

Another important fossil species known from the same Lagerstätte is *Santanmantis axelrodi* Grimaldi, 2003. Unlike *R. waddingtonae*, *S. axelrodi* is known from a number of specimens: nine specimens have been described in literature so far (Grimaldi, 2003; Grimaldi & Engel, 2005; Hörnig, Haug & Haug, 2013). The important phylogenetic position of the species within Mantodea as an early offshoot of the mantodean lineage was recognised early on (Grimaldi, 2003). Yet, the raptorial appendages were at first not well known (Grimaldi, 2003; Grimaldi & Engel, 2005). A not too well preserved additional specimen revealed that the raptorial appendages are already specialised with numerous prominent spines, including the prominent curved spine on the distal end of the tibia characterising Mantodea (Hörnig, Haug & Haug, 2013). Other morphological aspects of *S. axelrodi* appear more ancestral (plesiomorphic). This includes, for example, the comparably short and rather shield-like pronotum. Also most modern mantodeans have rather “naked” second and third pairs of thoracopods, i.e., lacking spines, while the condition in blattodeans and early dictyopterans is strongly spinose. Despite being not that well preserved, these appendages appeared to be plesiomorphically spinose in the single additional specimen of *S*. *axelrodi* (Hörnig, Haug & Haug, 2013). This led Hörnig, Haug & Haug (2013) to propose an evolutionary reconstruction termed “forelegs first”. It indicated that the stepwise acquisition of specialisations in the mantodean lineage first affected the first pair of thoracopods, i.e., specialising for raptorial purposes, while retaining a plesiomorphic morphology on the two pairs of posterior thoracopods. Then, in a second evolutionary step within Mantodea, the spineless two pairs of posterior thoracopods evolved as seen in modern forms. The morphology of *R*. *waddingtonae* is also in concordance with this suggestion (Dittmann et al., 2015). Yet, new findings of Cretaceous amber make the entire picture more complicated (Bai et al., 2016; see discussion further below).

We present here a new specimen of *S*. *axelrodi*. Its morphology indicates that the character evolution in the early lineage of Mantodea was in fact more complex than anticipated. We also discuss a new interpretation of the raptorial behaviour of *S*. *axelrodi* and early mantodeans in general.

## Material and Methods

### Material

The basis of this study is a single specimen on a slab from the famous Crato Formation (for further information about the Crato Formation see Martill, 2007 and Martill, Bechly & Loveridge, 2007). Material from the Crato Formation, which was formerly addressed as part of the Santana Formation (Martill, Bechly & Loveridge, 2007), is preserved in limestone. It was assigned to the Aptian (dated about 115 million years; Grimaldi, 2003, but see Martill, 2007; Martill, Bechly & Loveridge, 2007). The specimen is housed in the Museum für Naturkunde Berlin, Germany, under repository number MB.I.2068.

### Documentation method

The specimen was documented with a Canon EOS 70D equipped with a Canon MP-E 65 mm objective. Illumination was provided by Canon Twin Flash MT-24 equipped with polarisation filters. Light was cross-polarised to reduce reflections and enhance colour contrast. To overcome limited depth of field and limitations of field of view, several adjacent image stacks were recorded. To document the three-dimensional relief of the fossil, stereo pairs were recorded.

### Image processing

Image stacks were fused using CombineZM to achieve sharp images (Haug, Haug & Ehrlich, 2008; Haug et al., 2009; Haug et al., 2011a). Adjacent image details were fused using the photomerge function of Adobe Photoshop CS3 and 4 (Haug, Haug & Ehrlich, 2008; Haug et al., 2009; Haug et al., 2011a). Stereo pairs were assembled to stereo anaglyphs in Adobe Photoshop CS4 (Haug et al., 2010; Haug et al., 2011b; Haug et al., 2012). All images were optimised for histogram, saturation and sharpness in Adobe Photoshop CS4.

### Terminology

Terminology is kept on a rather general level to allow non-expert readers to follow. References to axes and directions of the appendages follow Hörnig, Haug & Haug (2013). Nomenclature of wing venation is based on Grimaldi (2003) for comparability.

**Figure 1:**
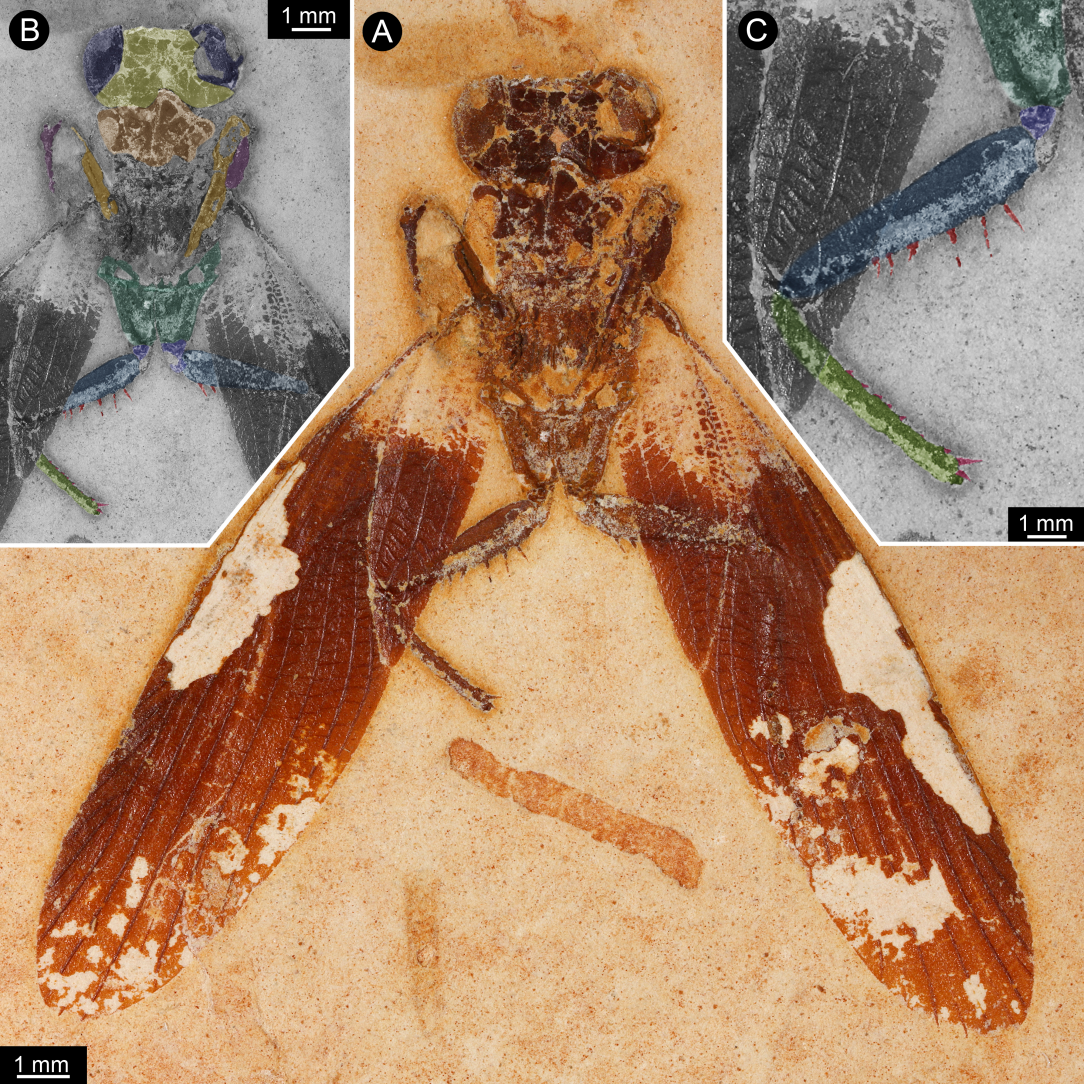
New specimen of *Santanmantis axelrodi* MB.I.2068. (A) Overview. (B) Colour-marked version of A; dark blue: eyes, yellow: head capsule, brown: tergite of thorax segment 1 (pronotum), orange: prothoracic femur, purple: prothoracic tibia, dark green: mesothoracic coxa, indigo: mesothoracic trochanter, blue: mesothoracic femur, light green: mesothoracic tibia, red: spines. (C) Detail of appendage of thorax segment 2, same colour code as in B.

**Figure 2:**
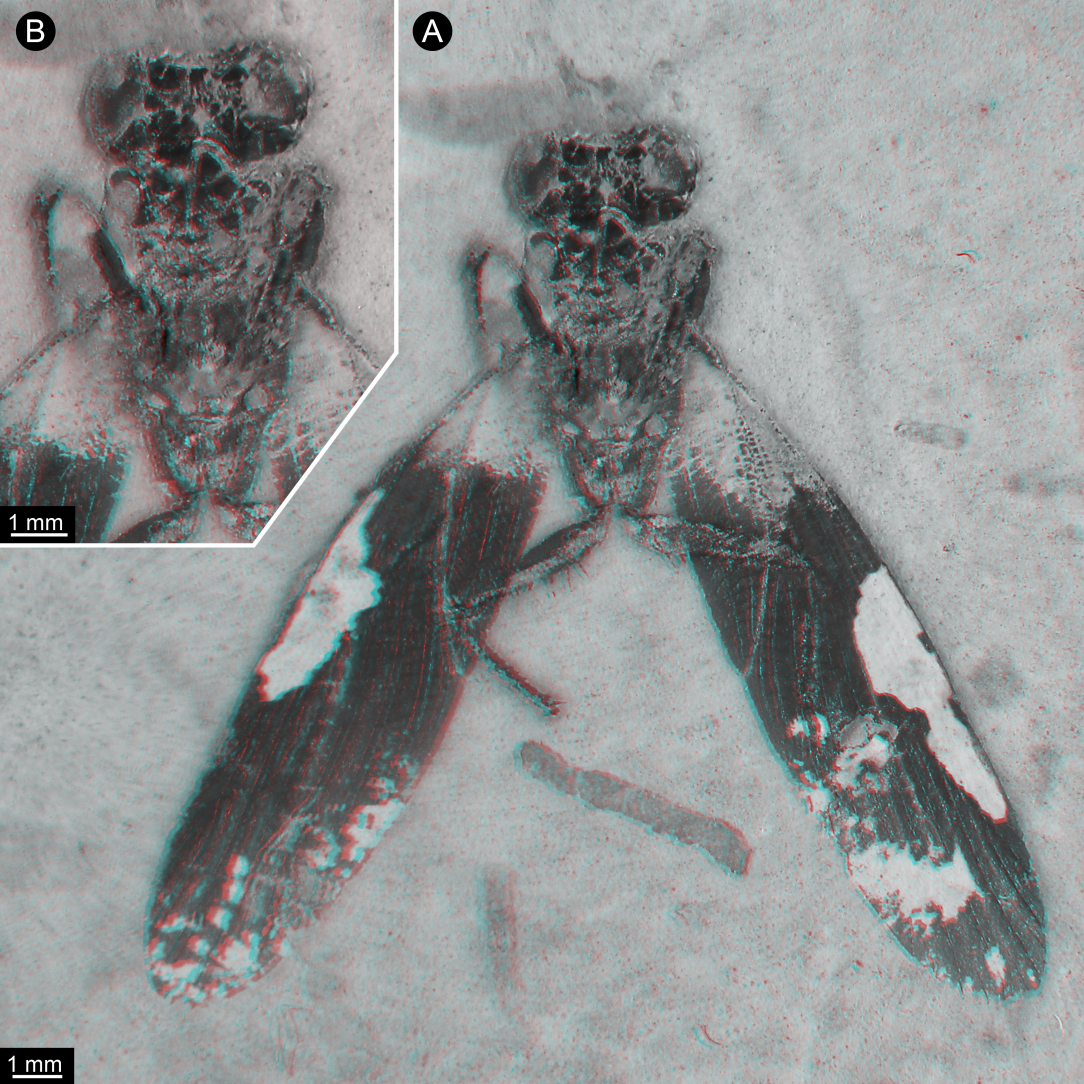
New specimen of *Santanmantis axelrodi* MB.I.2068, red-cyan stereo anaglyphs (use red-cyan glasses to view). (A) Overview. (B) Detail of main body, depth-inverted to show head in natural topology.

## Description of Specimen MB.I.2068

The specimen is preserved in dorsal view (Figs. 1 and 2). The preserved part of the body is organised into the head and the anterior part of the trunk (thorax, with prothorax and mesothorax). The posterior part of the trunk (metathorax and abdomen) is not preserved. Although preserved in dorsal view, many ventral details are accessible as some dorsal parts are missing, revealing the ventral ones. It is unclear whether the not preserved structures would be preserved on the counterpart.

The head is strongly set off from the trunk. It is wider (4 mm) than long (visible part 1 mm) with well apparent lateral eyes. These occupy the entire lateral region of the head. Further details such as sutures, insertions of antennulae (antennae), median eyes or similar are not identifiable. The lateral eyes do not show individual ommatidia (facets) preserved.

Of the thorax, mainly the appendages of the anterior two segments are preserved, as well as parts of the tergite of thorax segment 1 (pronotum), partly overhanging the head capsule, and postero-laterally extending wings of thorax segment 2 (mesothorax). Other parts of the mesothorax might be partly preserved, but not clearly definable due to preservation.

The first pair of thoracic appendages is tightly folded, with the tibiae occluding to the femora, therefore not much more details are accessible.

The second pair of thoracic appendages is well accessible. The left appendage is composed of four preserved elements. The most proximal element (coxa) is trapezoid/trapezium-shaped in anterior view and about 2.5 mm long. The second element (trochanter) is roughly triangular in anterior view with a gently rounded median edge and a length of about 0.4 mm. The third element (femur) is partly covered by the left forewing (distally, about one third of the length of femur), but the outline of the femur is compressed through the wing from below. The femur is more or less elongate rectangular in anterior view (most likely tube-shaped in original morphology), but with a swollen proximal region. The length of femur is about 3.3 mm, the maximum width is about 0.7 mm. Medially, the femur is armed with one row of 6 visible prominent spines, but as the femur is covered about one third by a forewing, more spines in this row are supposed for original condition. The spines are massive, quite long (longest spines preserved with a length of about 0.4 mm) and appear to possess a sharp tip and protrude from the femur in most cases at a distinct 90 degree angle. There is no clear indication that these spines were jointed and movable; in contrast, they widen at the base, indicating their general rigidity. There is no hind for further spines on the lateral or anterior surface of the femur. The fourth element (tibia) is also elongate tube-shaped and about 3 mm long. Spines are indicated along the entire median edge, yet broken off close to the base. Shape and length of these can therefore not be estimated. Distally a slightly curved medio-distally pointing spine is preserved, medially to the insertion of the tarsus. The tarsus is not preserved. On the right appendage of thorax segment 2 five elements are preserved. The two proximal elements (coxa and trochanter) are of the same morphology as on the left appendage. The third element (femur) is partly covered by the left forewing (distally, about two third of the length of femur), but the outline of the femur is compressed through the wing from below. The femur is more or less elongate rectangular in anterior view (most likely tube-shaped in original morphology), but with a swollen proximal region. The length of femur is about 3.3 mm, the maximum width is about 0.7 mm. Medially, the femur is armed with two visible prominent spines in one row, but as the femur is covered about two third by a forewing, the presence of more spines can be supposed originally. The spines are massive, quite long (about 0.3 mm preserved, but presumably broken off distally) and appear to possess a sharp tip. The fourth element (tibia) is completely covered by the right forewing, but partly visible in outline, which is compressed through the wing from below. The tibia is elongate tube-shaped and about 3 mm long. The tarsus is also covered by the wing, but partly visible. The tarsus is also elongate tube-shaped with a length of about 2.5 mm. Individual elements or more details of the tarsus are not visible.

**Figure 3:**
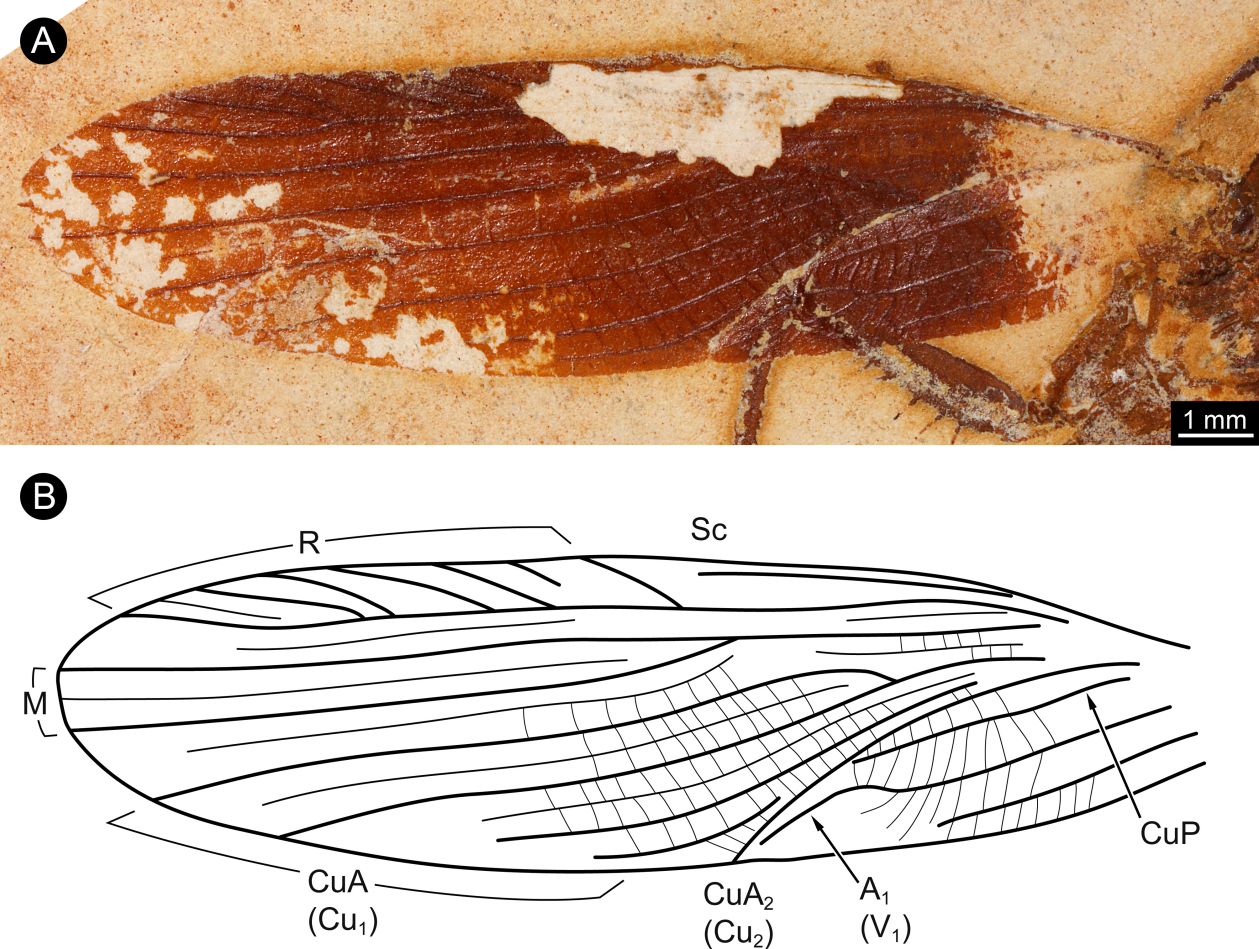
Details of wing of specimen MB.I.2068. (A) Close-up image of left wing. (B) Drawing of wing venation pattern. Nomenclature based on Grimaldi (2003) for comparability.

The wings of the mesothorax are well preserved (Fig. 3). They appear strongly sclerotised, representing tegmina. The length of wings is about 16 mm each, with a maximum width of about 4 mm medially. Sc vein is not completely preserved, most likely ends at about the half length of the wing. R with six main branches, CuA (Cu_1_) with four main branches (base not preserved). M splits at about the half of the length of the wing in two main branches. CuA_2_ (Cu_2_) is strongly curved. CuP is incomplete. A with two main branches, the last third part of left wing is presumably deformed by the underlying leg.

## Discussion

### Identification of the specimen

The specimen is identified as a representative of *Santanmantis axelrodi* based on its overall morphology. Similarities also include the venation of the preserved wings (Fig. 3). The specimen differs from other specimens interpreted as *S*. *axelrodi* in the clear presence of massive prominent spines on the femora and tibiae of the second pair of thoracic appendages. Yet, spines on legs appear to be regularly not preserved in other specimens of *S*. *axelrodi.* Only two specimens have provided the spines on the first pair of thoracic appendages (Hörnig, Haug & Haug, 2013). One of these two specimens and another specimen have indicated the mere presence of spines on the second pair of thoracic legs, but no clear details of their morphology (Grimaldi, 2003; Hörnig, Haug & Haug, 2013).

This difference (presence of massive spines) of the new specimen and older material is therefore interpreted as preservational, not representing a diagnostic character for delineating a new species. We assume that they are simply not preserved in other specimens.

As a further difference the new specimen is comparably large. Grimaldi reported forewing lengths for specimens of *S. axelrodi* of between 11–13 mm. The here described specimen has a significantly larger wing length of 16 mm. Yet, such size differences are comparable to the size variations among modern mantodeans. For example, the length of the prothorax of females of *Stagmomantis limbata* Hahn, 1835 varies at least from 17–24 mm (Maxwell & Frinchaboy, 2014). Males are significantly smaller. Therefore, we interpret the new specimen as a quite large (female?) representative of *Santanmantis axelrodi.*

### Updated reconstruction of *S. axelrodi*

The new specimen provides new details of the morphology of the second pair of thoracic appendages (mesothoracic legs). Coxa, trochanter, femur and tibia are nearly completely preserved.

**Figure 4:**
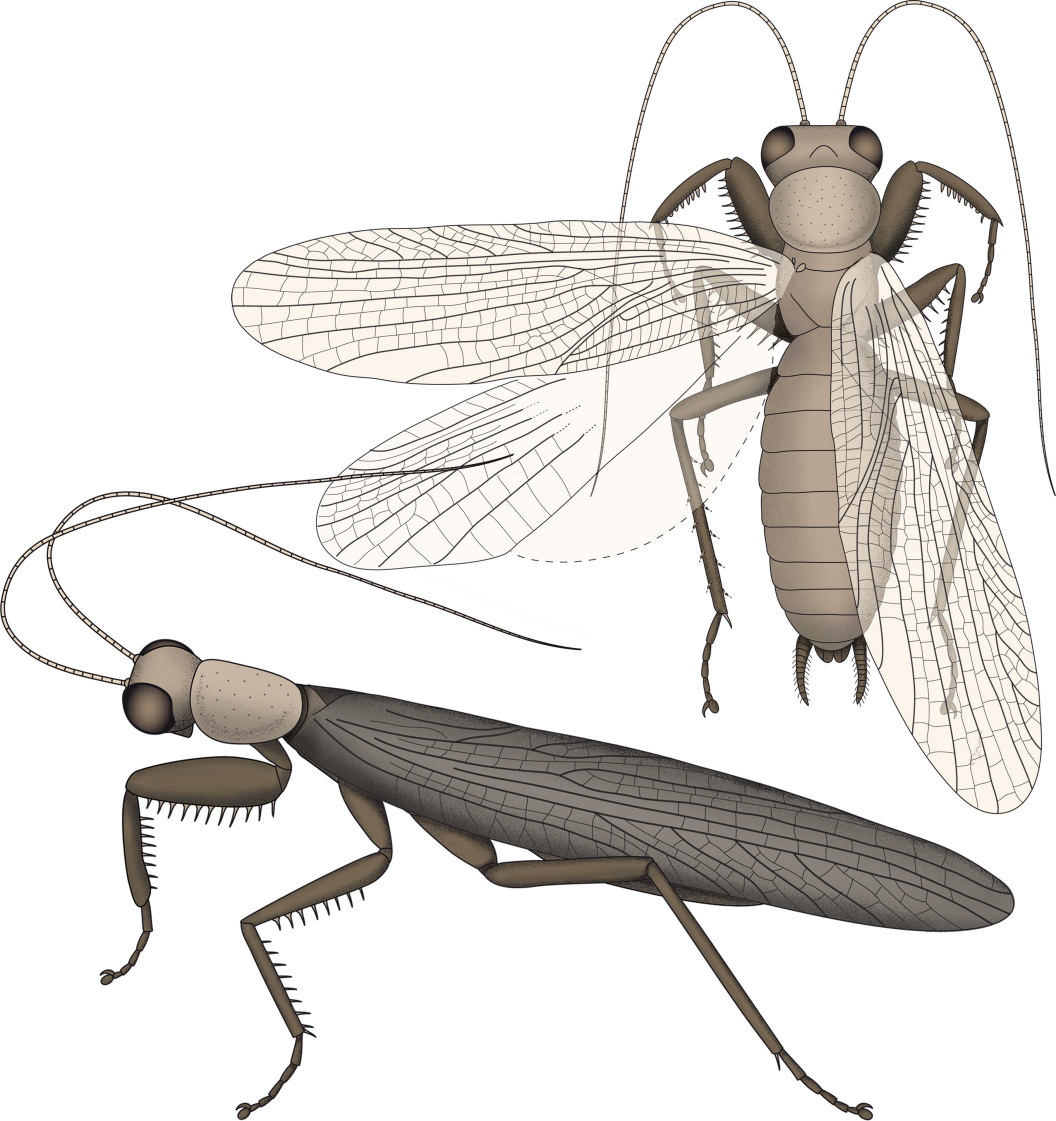
Restorations of *Santanmantis axelrodi* based on Hörnig, Haug & Haug (2013), amended by the newly discovered details.

The new specimen indicates that all other specimens of *S. axelrodi* have an incomplete preservation concerning the armature of the second pair of thoracic appendages. We therefore have to amend the reconstruction of Hörnig, Haug & Haug (2013), which already represents an amended version of the reconstruction of Grimaldi (2003) (Fig. 4).

As already known previously, *S*. *axelrodi* is an early mantodean with a highly specialised mantodean-type spinose first pair of thoracic appendages. Although the presence of spines on the first and second pair of thoracic appendages was indicated based on two specimens (Grimaldi, 2003; Hörnig, Haug & Haug, 2013), the preservation of the second thoracic leg is only fragmentary on these fossils.

The new specimen clearly shows that at least the femur of the second pair of thoracic legs is medially armed with massive and quite long spines that stand erect, perpendicular to the main appendage axis. Previous reconstructions included short movable and obliquely oriented spines. Instead, these spines resemble the spines on the femur of the first pair of thoracic appendages in rigidity, shape, length, orientation and pointedness.

We could speculate that this type of morphology was also present on the third pair of thoracic appendages. Yet, as it is not well preserved in the specimen at hand this must remain unclear. Given the fact that out of now ten specimens of *S*. *axelrodi* only three have fragmentarily preserved spines on different parts of appendages, it could well be possible that another specimen might reveal similar structures on the third pair of thoracic appendages in the future.

An important new observation concerns the relative lengths of the leg elements of the thoracic appendages. Grimaldi (2003) interpreted the tibia to be longer than the femur in the second pair of legs (in two of his specimens where this is accessible). This is different in the new specimen, where the femur is longer than the tibia. This latter condition is also found in all modern forms. Yet, in modern forms the tibia is longer than the femur in the third pair of appendages. We therefore see it as likely that the appendages interpreted as mesothoracic by Grimaldi (2003) in fact represent the metathoracic appendages. This would represent a more consistent interpretation.

### Implications for the character evolution within Mantodea

**Figure 5:**
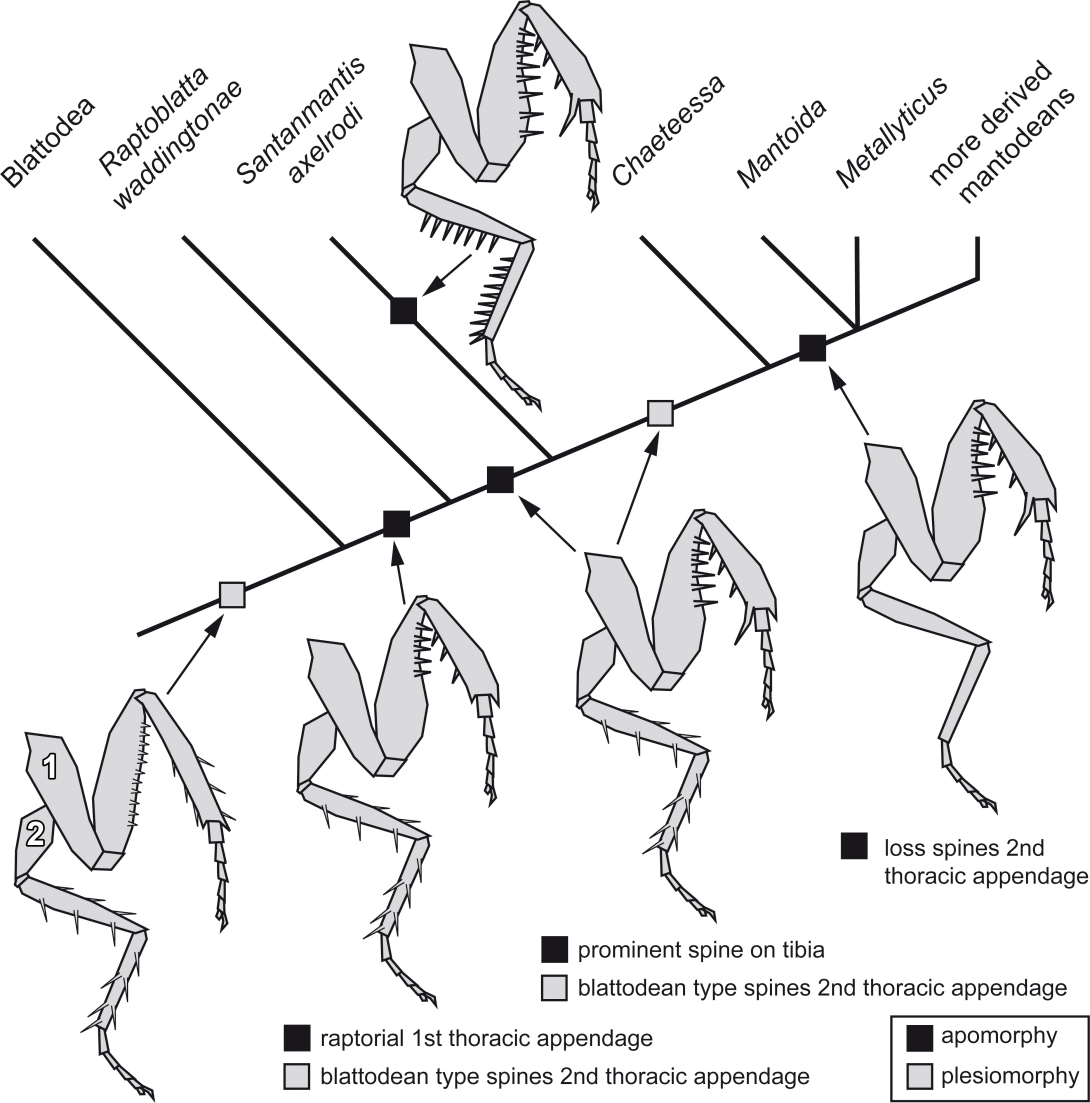
Possible reconstruction of character evolution within the mantodean lineage. 1: first thoracic appendage; 2: second thoracic appendage.

The new morphological details of *S. axelrodi* influence the reconstruction of character evolution within Mantodea (Fig. 5). Before, the known specimens showed in principle two conditions of the mesothoracic appendages:

– Condition one is seen in blattodeans: femur and tibia are often strongly spinose; the spines on the femur are comparably short, appear blunt and are usually movable to a certain extent (Bohn, 2003; Wieland, 2013). On the tibia the often more pronounced spines are arranged uniformly around the leg.
– Condition two is seen in modern mantodeans: femur and tibia lack prominent armature (Wieland, 2013). An exception of this is the mantodean ingroup *Chaeteessa.* Representatives of this group bear distinctly articulated (i.e., movable) spines on second and third pair of thoracic appendages. Based on the structure of the spines and their overall similarity to blattodean type spines, this has been interpreted as a retained plesiomorphic condition (Wieland, 2013).

For many fossil representatives of Mantodea known so far, we can unfortunately not clearly identify which condition is present due to preservational aspects. As the former specimens of *S*. *axelrodi* provided indications of presence of spines on the second pair of thoracic appendages, these were interpreted as condition one (Hörnig, Haug & Haug, 2013). Condition two would then have evolved within Mantodea (“forelegs first” hypothesis of Hörnig, Haug & Haug (2013). The new specimen now clearly represents a previously unknown third condition, with erect immovable prominent pointed spines. (The reconstruction in Hörnig, Haug & Haug (2013) interprets the morphology of the mesothoracic appendages in the text as condition one, but erroneously depicts already an indication of condition three.)

The early fossil record of the mantodean lineage has provided numerous further species of Cretaceous age. Unfortunately, many of these fossils contribute only little to the discussion of character states of trunk appendage details. For example, the well preserved holotype of *Ambermantis wozniaki* Grimaldi, 2003 provides an exceptionally preserved first thoracic appendage (strongly resembling modern mantodeans; Grimaldi, 2003), but the distal parts of the second appendage were outside the resin during formation and are hence not included in the amber. The third pair of thoracic appendages lacks armature, possibly indicating condition two.

For many other fossils in amber such as *Burmantis asiatica* Grimaldi, 2003, *B. zherikhini* Delclòs et al., 2016, or *Chaeteessites minutissimus* Gratshev & Zherikhin, 1993 likewise details of the prothoracic appendages are available, while we lack detailed access to the mesothoracic ones (Grimaldi, 2003; Delclòs et al., 2016). *Jersimantis burmiticus* Grimaldi, 2003 is an exception providing details of prothoracic and mesothoracic appendages, the latter ones with condition two. A newly published specimen of a nymph of *B. lebanensis* Grimaldi, 2003 by Delclòs et al. (2016) which is nearly complete preserved, is described with no spination of the femur (but setae in the drawing?) and two large spurs distally and short spine-like setae on the tibia of the mesothoracic legs. The tibial spine-like setae are described to be arranged in one row, but based on the photographs and drawing of the specimen we interpret these as arranged uniformly around the tibia. *B. lebanensis* can be also interpreted as representative of condition two.

The single specimen of *Cretomantis larvalis* Gratshev & Zherikhin, 1993 is preserved on a slab and appears to have born spines on the mesothoracic appendages, yet their exact nature is unclear (Grimaldi, 2003). In many instances spines appear to be broken off, preserving only the bases, with this hindering a clearer statement for the condition in this species. In some areas one could think that there is an indication for a similar morphology as in *S. axelrodi*, but this remains difficult to judge. Given the proposed possible close relationship of *S. axelrodi* and *C. larvalis* (Grimaldi, 2003) this character could indeed unite the two.

The recently described *Alienopterus brachyelytrus* Bai et al. (2016) has been proposed to represent the sister group to Mantodea (although not considering the relative position of *R. waddingtonae*). The legs of this species differ from the condition in other dictyopterans in bearing numerous fine setae, which most likely represents an apomorphic condition of this species (Bai et al., 2016).

How can we incorporate the newly observed condition into the reconstruction of character evolution within Mantodea? A single extant mantodean group is known with representatives bearing spines on the meso- and metathoracic appendages, *Chaeteessa* (e.g., Gratshev & Zherikhin, 1993; Wieland, 2013, p. 91). This spination resembles in structure and arrangement the condition in extant cockroaches or *Jersimantis burmiticus*, but not that of *S. axelrodi* (and possibly that of *C. larvalis*). We see it therefore as most likely that the condition of *S. axelrodi* represents either an autapomorphy of the species or a larger group possibly also including *C. larvalis*. The ground pattern condition of the metathoracic appendage for Mantodea is most likely similar to the morphology in *Chaeteessa*. The “forelegs first” hypothesis therefore seems valid. Yet, the case of *S. axelrodi* demonstrates that within the early lineage of Mantodea more variation concerning this character occurs.

### Remaining questions in character evolution

While the discussion of character transformation in the leg spination is informative, it also confronts us with a major challenge: incomplete knowledge. The condition is unclear for many of the early branches of Mantodea, we have therefore not included many of the other early branches in our reconstruction. This is not unusual and in many aspects unfortunate, but reflects our current knowledge.

This also concerns other aspects of the evolutionary reconstructions. We support the view of Dittmann et al. (2015) that *Raptoblatta waddingtonae* shares important characters with Mantodea, all related to the raptorial appendages. Bai et al. (2016) suggested that *Alienopterus brachyelytrus* should represent the sister species to Mantodea (not discussing *R. waddingtonae*), but based on characters of the genitalia. Can this issue be resolved? *A. brachyelytrus* has a prothoracic appendage with a slightly shortened tibia; also a demarcated area on the femur in which the tibia could be folded seems to be morphologically specialised (Bai et al., 2016, their Fig. 3A). Still, the femur lacks the specialised shape seen in mantodeans and *R. waddingtonae*, as well as the specialised spination. A possible interpretation is therefore that *A. brachyelytrus* is the sistergroup to *R. waddingtonae* + Mantodea. Yet, this would predict that *R. waddingtonae* would already have possessed the asymmetric genitalia seen in males of Mantodea and *A. brachyelytrus*. This can currently not be tested, but would suggest that the specialised genitalia evolved before the highly modified raptorial appendages.

Relationship within the lineage towards modern mantodeans will still need to see further considerations. Yet, there is no scientific value in trying to break this lineage into separate orders (see discussion about the general problems with ranks in Satoh, Rokhsar & Nishikawa, 2014, Lambertz & Perry, 2015, Lambertz & Perry, 2016 and Giribet, Hormiga & Edgecombe, 2016). Careful and detailed consideration of characters in an evolutionary framework is necessary. This includes the problem of recognising differences as possibly being related to biology. For example, the new insect order erected by Poinar Jr & Brown (2017) seems likely based on immature specimens of *A. brachyelytrus* and demands for a reinvestigation of this material (see Haug & Haug, 2016 for a general discussion of this issue).

### Raptorial behaviour in the early mantodean lineage

The newly observed morphological details have consequences for the interpretation of the capture behaviour of *S. axelrodi*. For a better understanding of this aspect, we need to compare our findings to extant species, as we have, of course, no direct observations of behavioural aspects of extinct mantodeans.

The mantodean ingroups *Metallyticus*, *Chaeteessa* and *Mantoida* are generally interpreted as the earliest branches of Mantodea with extant representatives. Representatives of all these three groups possess a short prothorax. This has been assumed to be related to their specific habitat on tree trunks or under loose bark of trees (habitat known for *Metallyticus*, assumed for *Chaeteessa* and *Mantoida*; Wieland, 2013). Such a condition could possibly be assumed for early mantodeans in general and as well for *S. axelrodi*.

Wieland (2013) assumes a functional correlation between length of prothorax and length of coxae and femora of the first pair of thoracic appendages. An extreme elongation of coxae and femora of the prothoracic appendages should be only possible if the prothorax is elongated as well, as the coxa-femur joint would otherwise interfere with the second pair of thoracic appendages. Furthermore, he suggested that it is necessary that the mouthparts of the mantodean reach to the area between the spines on femur and tibia of the prothoracic appendages (Wieland, 2013).

Concluding further, an elongation of the raptorial appendages (in relation to body size) and prothorax became “useful” when mantodeans changed their lifestyle from bark dweller to a more open habitat. While the majority of modern mantodean species has been considered to be ambush predators, representatives of *Metallyticus* are active hunters (Wieland, 2008). This can also be assumed for other early mantodeans and also *S. axelrodi*.

Early modern mantodeans, species of *Metallyticus*, *Chaeteessa* and *Mantoida*, also differ in another aspect from other mantodean species. While resting, the tarsi of the raptorial legs are placed on the ground. This was assumed to represent the plesiomorphic condition for Mantodea by Wieland (2013). In both specimens of *S. axelrodi* with preserved first pair of thoracic appendages (Grimaldi, 2003; Hörnig, Haug & Haug, 2013), femur and tibia are folded against each other. This can be a preservational effect, but most of the second and third pairs of thoracic appendages are preserved in extended position. We could therefore suggest that *S. axelrodi* had folded the first pair of legs against the prothorax in a resting position. This results in a complex character pattern which can currently not be resolved.

Further behavioural aspects, including prey capturing, is partly known for representatives of *Metallyticus*, but not for *Mantoida* and *Chaeteessa*. Unfortunately, *Metallyticus* is characterised by a highly derived and specialised pair of raptorial appendages with an extremely elongated spine on the proximal part of the femur, as well as complete lack of spination of the second and third pair of thoracic appendages (Wieland, 2008; Wieland, 2013). Therefore, the raptorial behaviour of representatives of *Metallyticus* is hardly comparable to that of *S. axelrodi.* Still the general way of locomotion (e.g., running, approaching to prey) could be similar based on a comparable general habitus (short prothorax, rather flat body, comparable length of appendages in relation to the body).

### Implications for the raptorial behaviour of *S. axelrodi*

The newly observed structures indicate a rather different raptorial behaviour of *S. axelrodi* than seen in modern mantodeans. The spines of the second pair of thoracic appendages strongly indicate that these appendages were involved in the prey-catching process. Similar erected pointed spines on raptorial appendages are known in numerous arthropods including extreme examples such as amblypygid whip spiders (Weygoldt, 2000), extinct thylacocephalan crustaceans (Haug et al., 2014 and references therein), emesine heteropterans (Wygodzinsky, 1966) and laniatore harvestmen (Pinto-da Rocha, Machado & Giribet, 2007). If the second pair of thoracic appendages was indeed involved in capturing prey, i.e., for immobilising the prey, an individual of *S*. *axelrodi* must have gathered a position almost covering the prey.

In most insects that perform raptorial behaviour with their thoracic appendages only the first pair is used. This accounts not only for modern mantodeans, but also, for example, for mantispid neuropterans (Eggenreich & Kral, 1990) or some groups of heteropterans (e.g., Emesinae, Belostomatidae, Nepidae; Wygodzinsky, 1966; Grimaldi & Engel, 2005; Weirauch, 2006). Further posterior appendages are rarely involved. Notable exceptions are (1) mantophasmatodeans (Roth, Molina & Predel, 2014), (2) predatory katydids (e.g., Marshall & Hill, 2009, their Fig. 7A; Fialho et al., 2014, their Fig. 9), and (3) odonatans (St. Quentin, 1953; Leipelt, Suhling & Gorb, 2010):

(1) Mantophasmatodeans differ from *S. axelrodi* in spine length. The spines of *S. axelrodi* are significantly longer than those of mantophasmatodeans. Additionally, mantophasmatodeans have a very flexible body axis (Roth, Molina & Predel, 2014), which certainly influences their entire motions, including prey catching. It cannot be assumed that this condition is also the case in *S. axelrodi*, as this extreme flexibility of the body has not been reported for any dictyopteran.
(2) Raptorial katydids differ significantly from *S. axelrodi* in their size. Katydids are super-sized insects, much more massive than *S. axelrodi*. This makes raptorial katydids also not a useful comparison.
(3) Odonatans use their raptorial appendages for catching prey while flying. Odonatans are exceptional flyers, while dictyopterans are generally not regarded as good flyers. It seems therefore unlikely that *S. axelrodi* caught prey in a comparable manner to odonatans.

As a summary we do not seem to have a good comparison among modern insects to *S. axelrodi*. We can therefore only assume that the second pair of thoracic appendages somehow immobilised the prey, but not directly grabbed it. Further details can currently not be reconstructed.

### Indications for the raptorial behaviour in other early mantodeans?

So, if the raptorial behaviour of *S*. *axelrodi* differs from that of modern mantodeans, as well as from that of other insect groups, what does this indicate for the raptorial behaviour in other early mantodeans? The early representatives of the mantodean lineage must have caught prey in a different way than most modern mantodeans. This is especially coupled to the elongate prothorax in most modern forms, which is still very short in early mantodeans. So far, no mantodean fossil from the Cretaceous is known with a prominently elongate prothorax (Grimaldi, 2003; Bai et al., 2016; see summary in Wieland, 2013). In *R*. *waddingtonae,* a raptorial representative of Cretaceous dictyopterans, the pronotum still has partly covered the head, similar to other non-mantodean dictyopterans. We can therefore assume that is was not able to lift the anterior body and perform a downward oriented strike as seen in modern mantodeans.

Raptorial behaviour in the mantodean lineage appears to be less stereotypic than it might be assumed from modern derived forms. Early offshoots of the lineage must have employed quite different types of movements than modern forms with elongated first thoracic segments. Representatives of the recently described *A. brachyelytrus* possess only few adaptations of the raptorial legs shared with mantodeans and *R. waddingtonae*, but are specialised on their own in having a dense surface armature with small setae. Together with *S*. *axelrodi* all these finds demonstrate that early representatives of the lineage appear to have “experimented” with different strategies. This is also indicated by the fact that the spination of the first pair of thoracic appendages of *S. axelrodi* differs from that of *R. waddingtonae* and other mantodeans (Dittmann et al., 2015). Also the “primitive” modern forms still show a certain variation: representatives of *Metallyticus* have a highly specialised raptorial appendage and representatives of *Chaeteessa* (secondarily) lack the prominent spine on the tibia. The modern types of behaviour are likely to be a kind of a left-over of an originally more diverse set of different prey-catching behaviours.

##  Supplemental Information

10.7717/peerj.3605/supp-1 Supplemental Information 1 Single images of image stacks and stereo

## Additional Information and Declarations

### Data Availability

The following information was supplied regarding data availability: The raw data has been provided as a Supplemental Information 1.
