# Supplementary figures and images for: An exceptionally preserved 110 million years old praying mantis provides new insights into the predatory behaviour of early mantodeans
Source: PeerJ. 2017 Jul 24;5:e3605. doi: 10.7717/peerj.3605 (PMC5527957; doi:10.7717/peerj.3605)

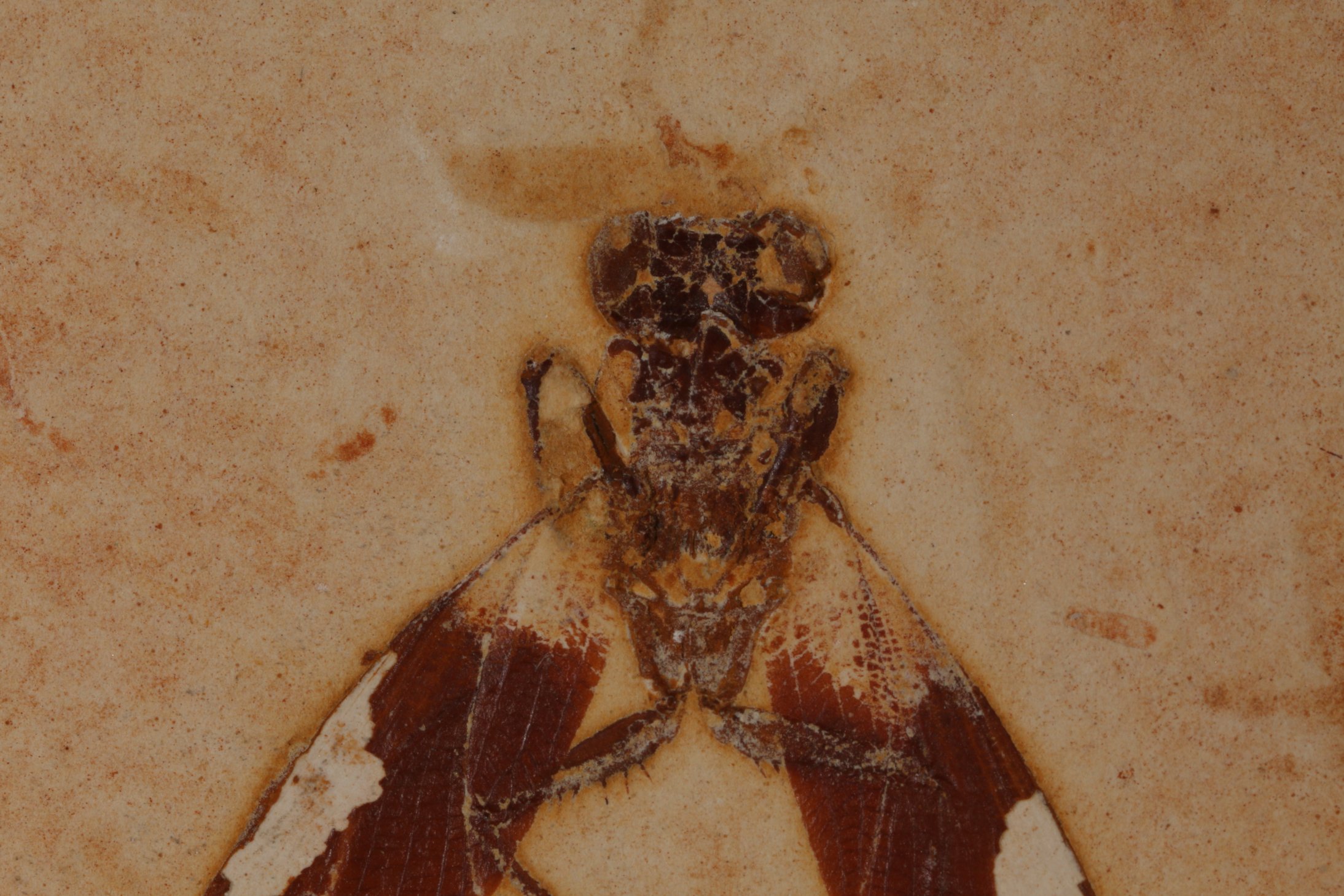

Supplement: Supplemental Information 1 [file peerj-05-3605-s001.zip › Santanmantis2_rawdata0000.jpg]

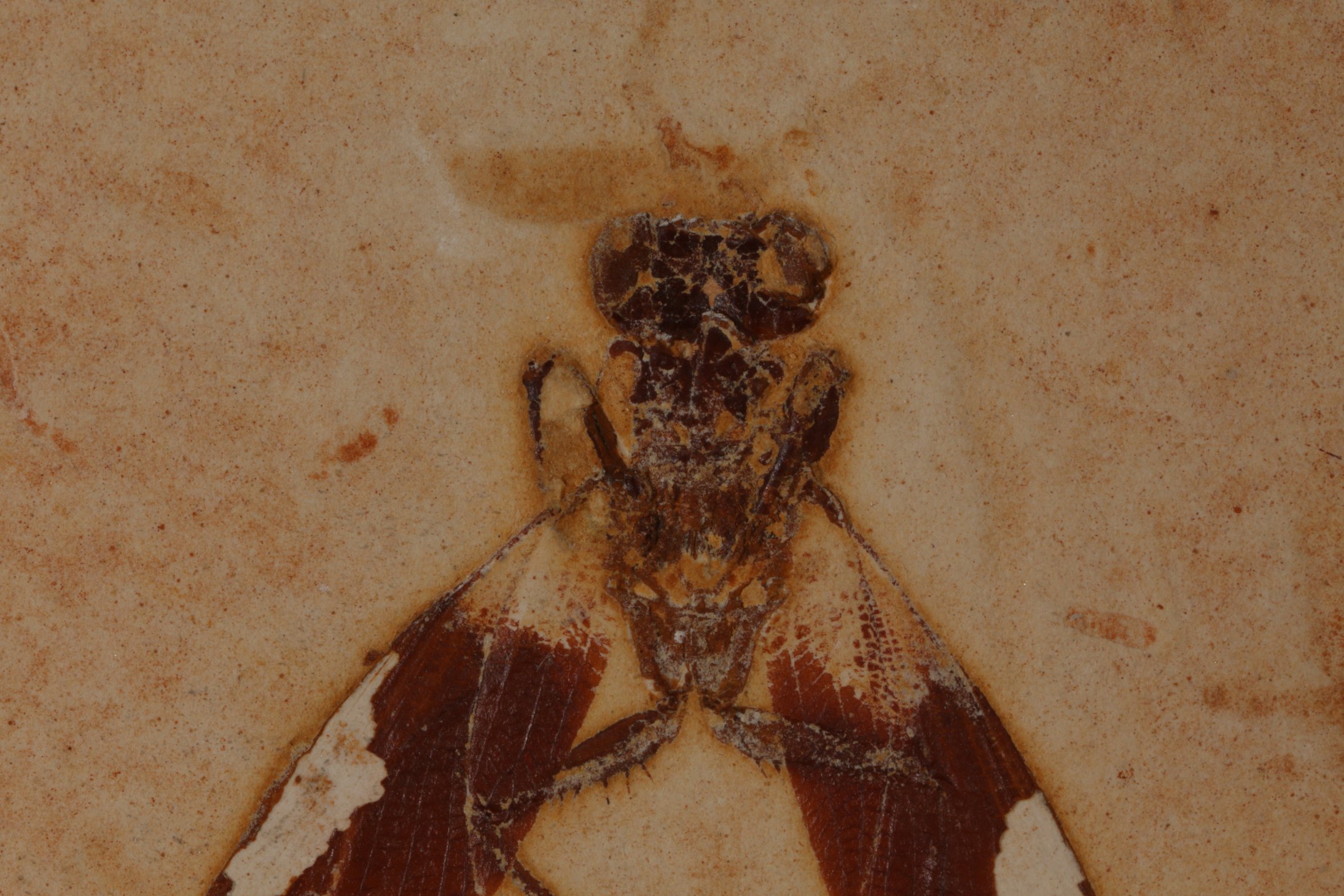

Supplement: Supplemental Information 1 [file peerj-05-3605-s001.zip › Santanmantis2_rawdata0001.jpg]

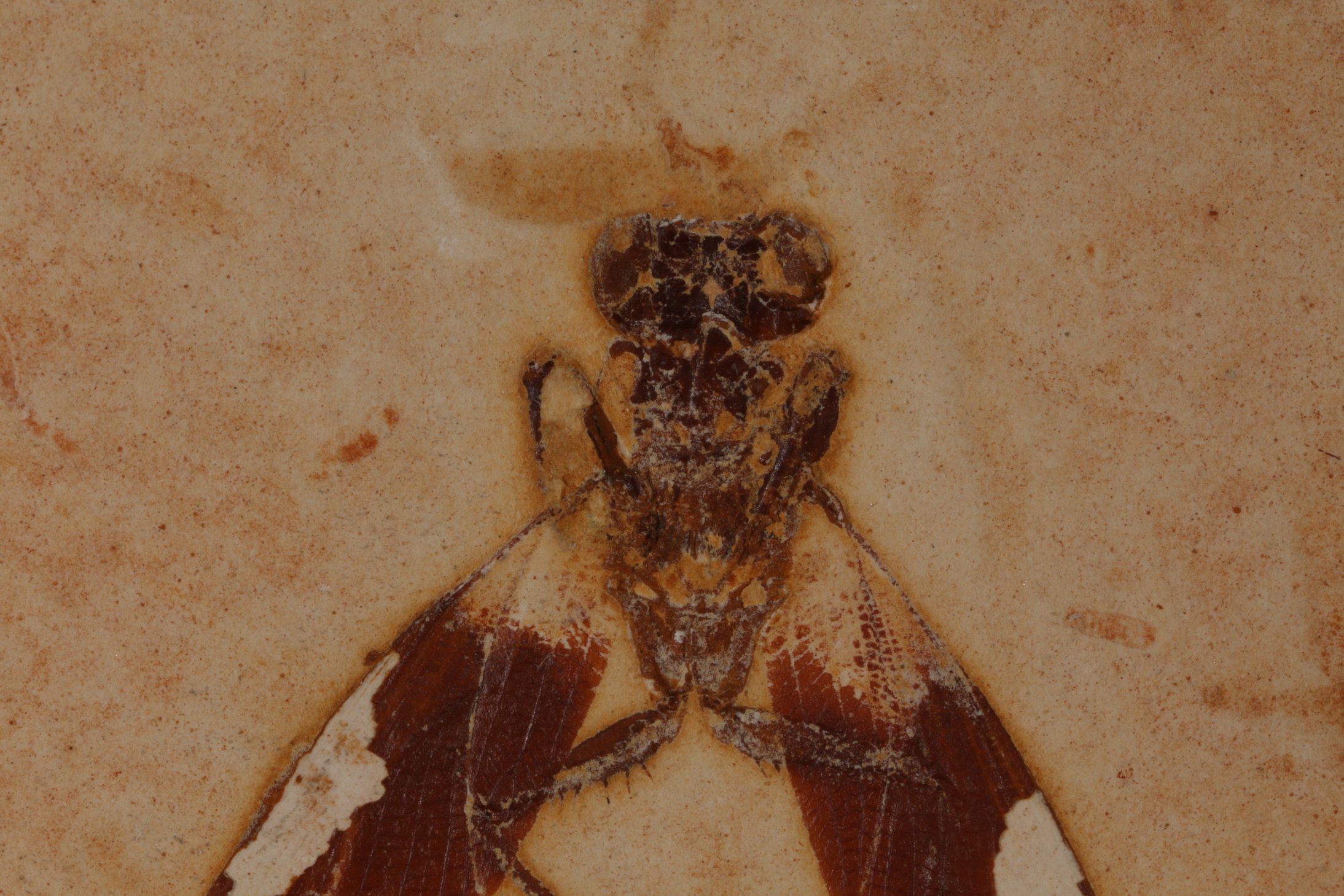

Supplement: Supplemental Information 1 [file peerj-05-3605-s001.zip › Santanmantis2_rawdata0002.jpg]

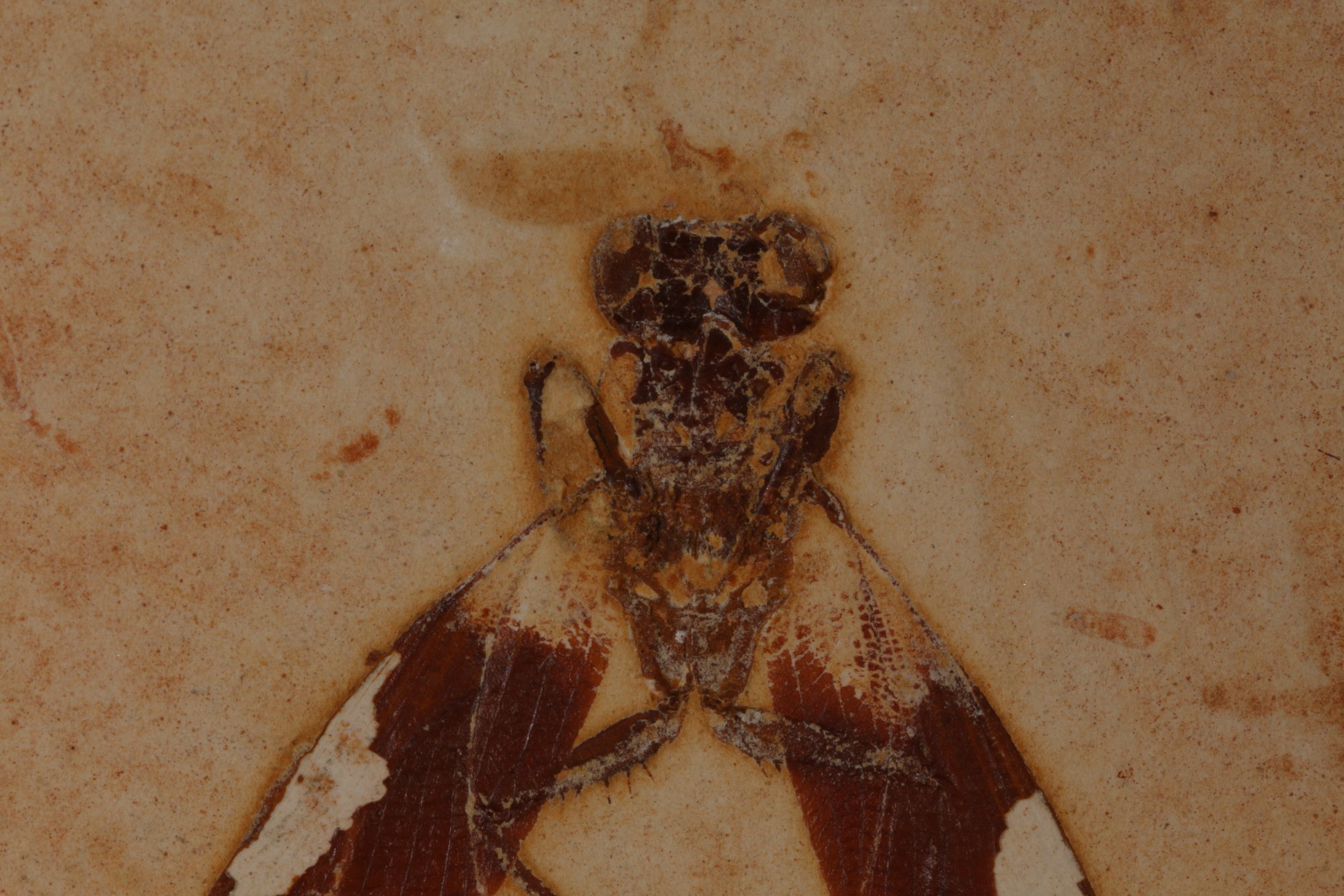

Supplement: Supplemental Information 1 [file peerj-05-3605-s001.zip › Santanmantis2_rawdata0003.jpg]

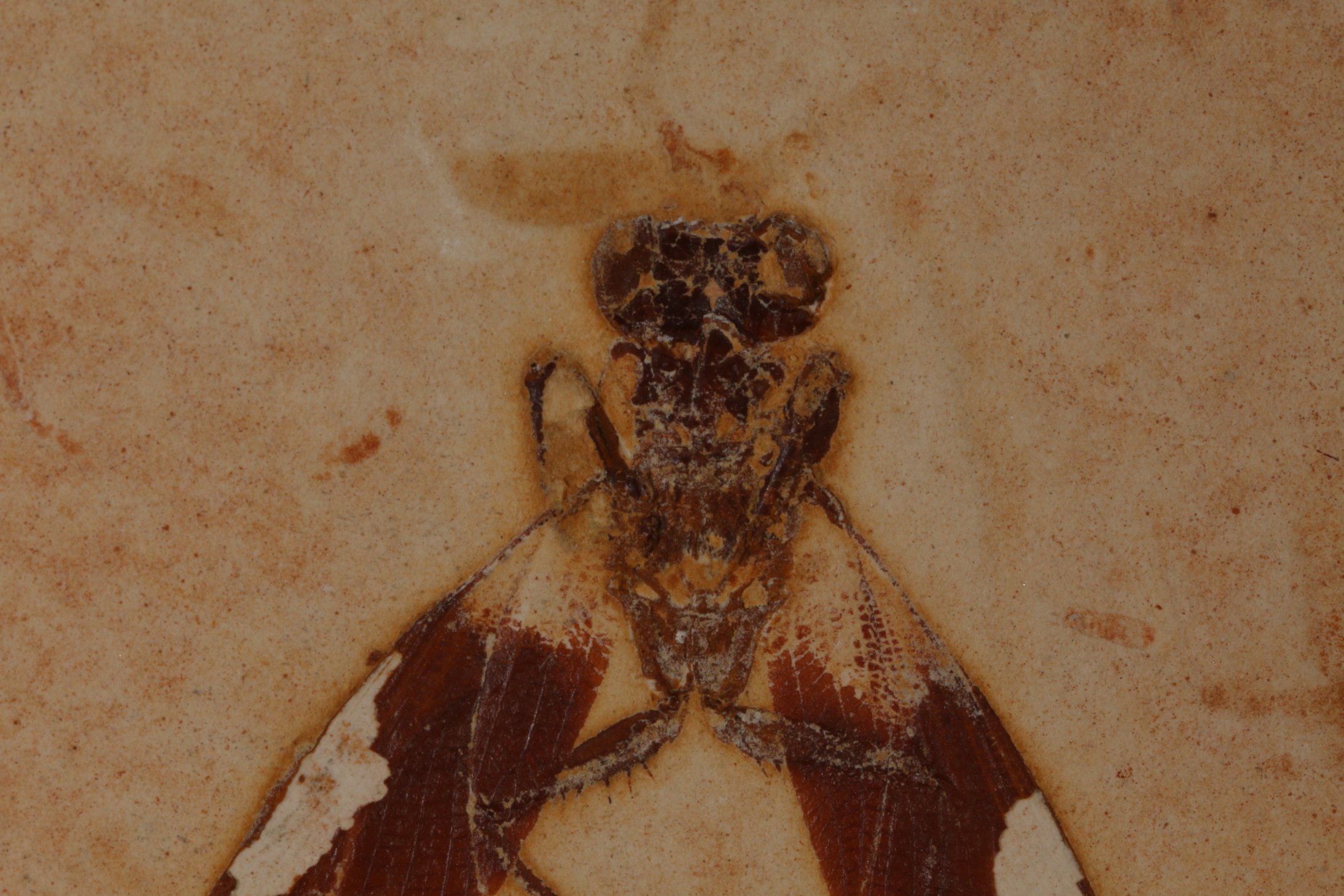

Supplement: Supplemental Information 1 [file peerj-05-3605-s001.zip › Santanmantis2_rawdata0004.jpg]

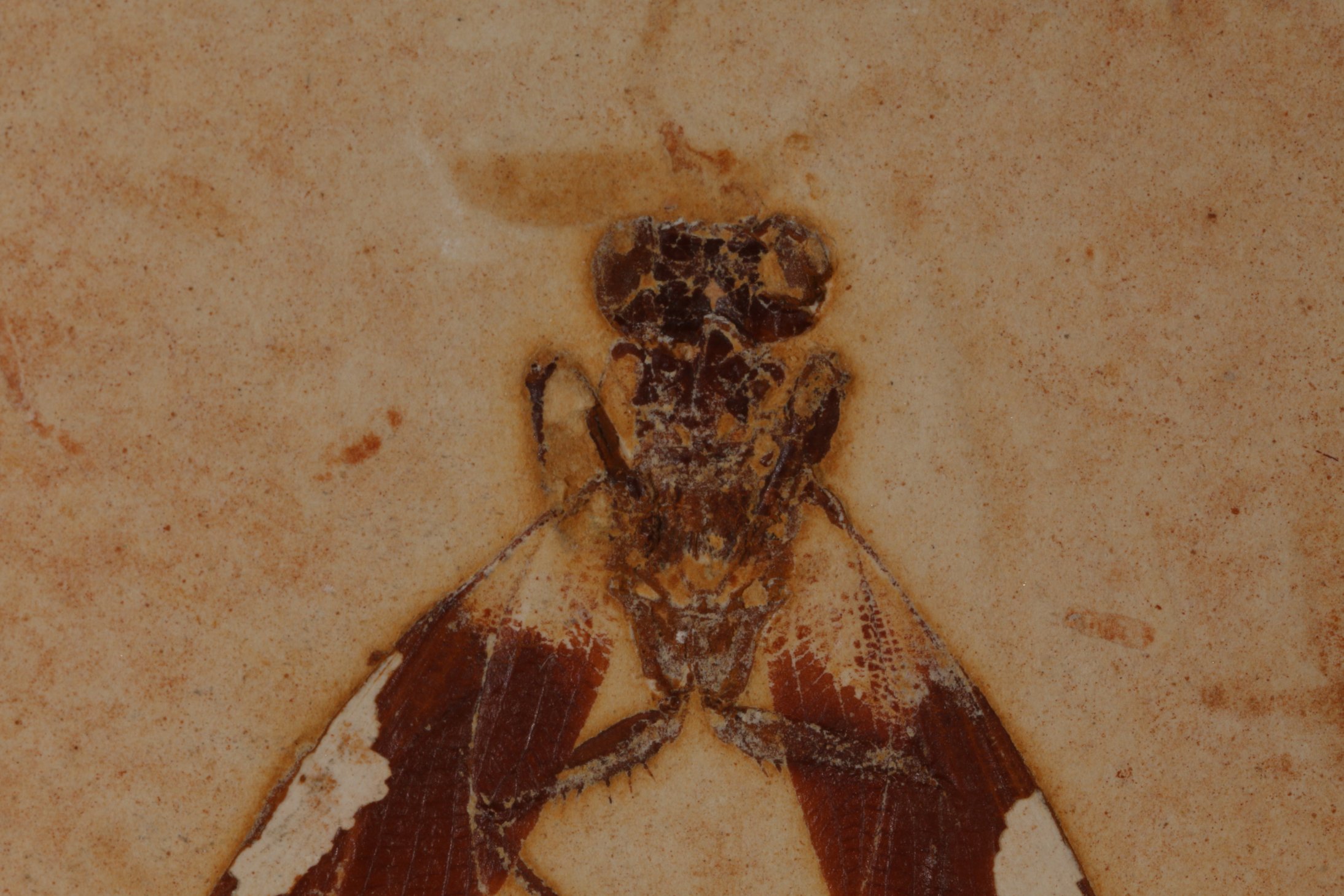

Supplement: Supplemental Information 1 [file peerj-05-3605-s001.zip › Santanmantis2_rawdata0005.jpg]

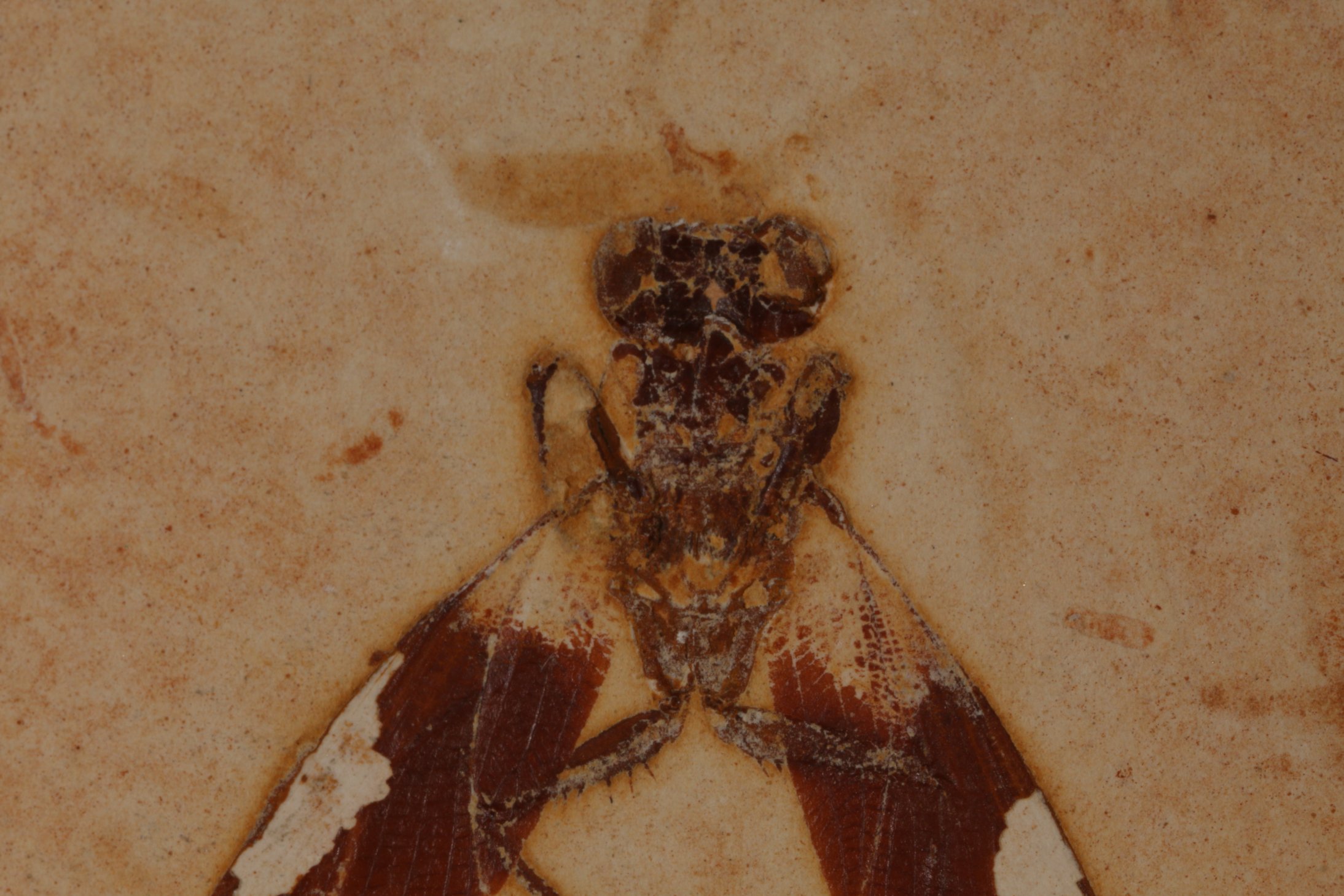

Supplement: Supplemental Information 1 [file peerj-05-3605-s001.zip › Santanmantis2_rawdata0006.jpg]

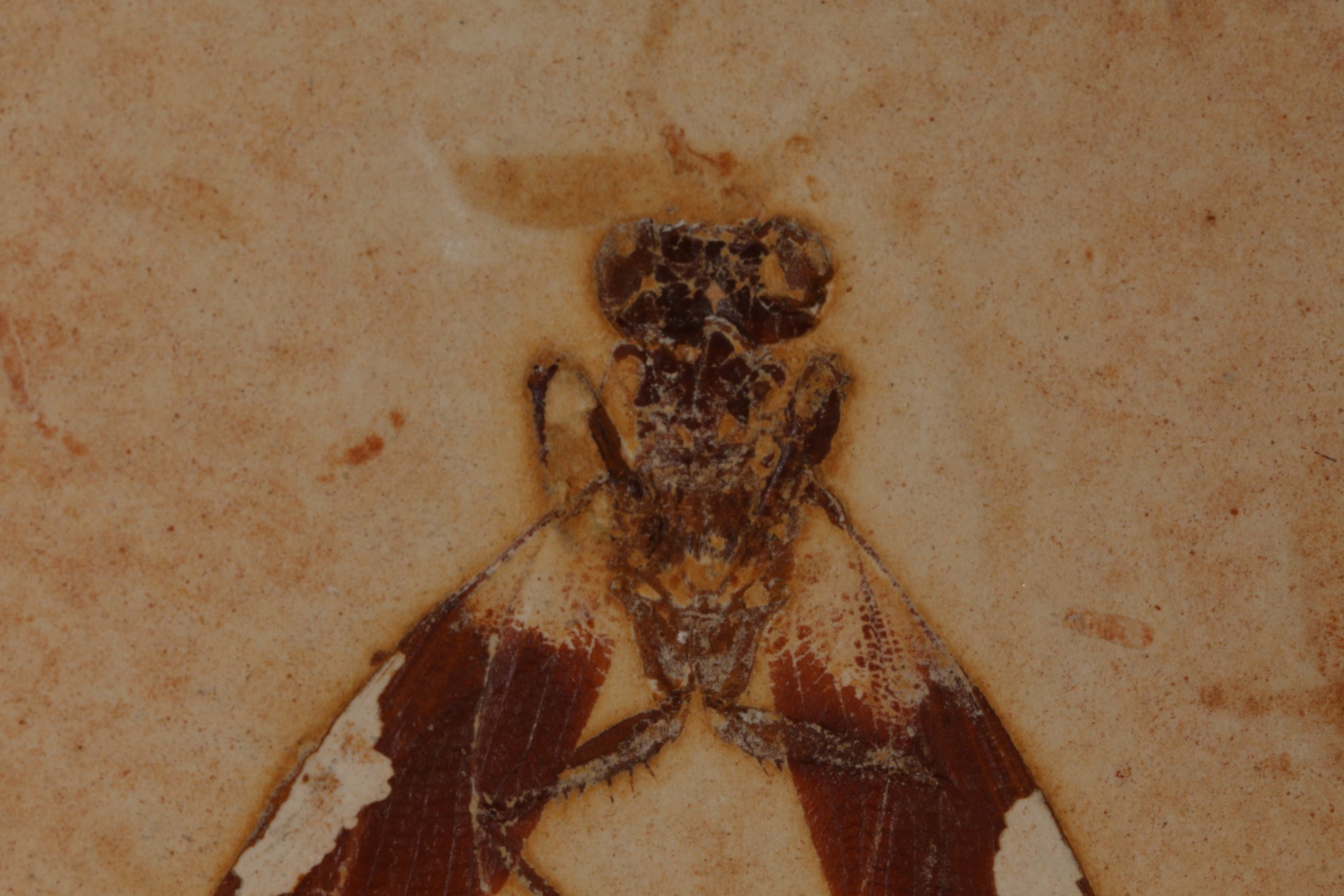

Supplement: Supplemental Information 1 [file peerj-05-3605-s001.zip › Santanmantis2_rawdata0007.jpg]

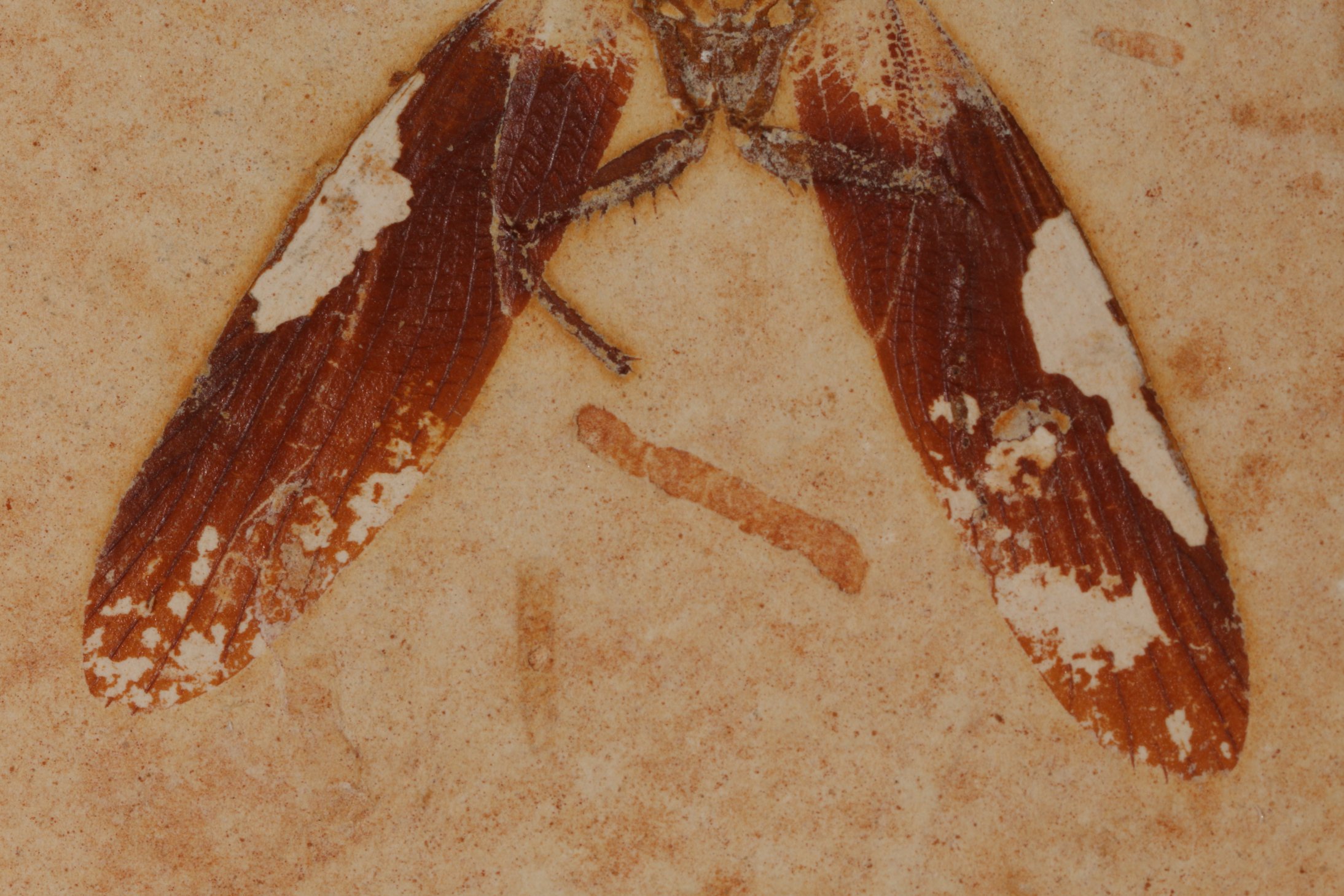

Supplement: Supplemental Information 1 [file peerj-05-3605-s001.zip › Santanmantis2_rawdata0008.jpg]

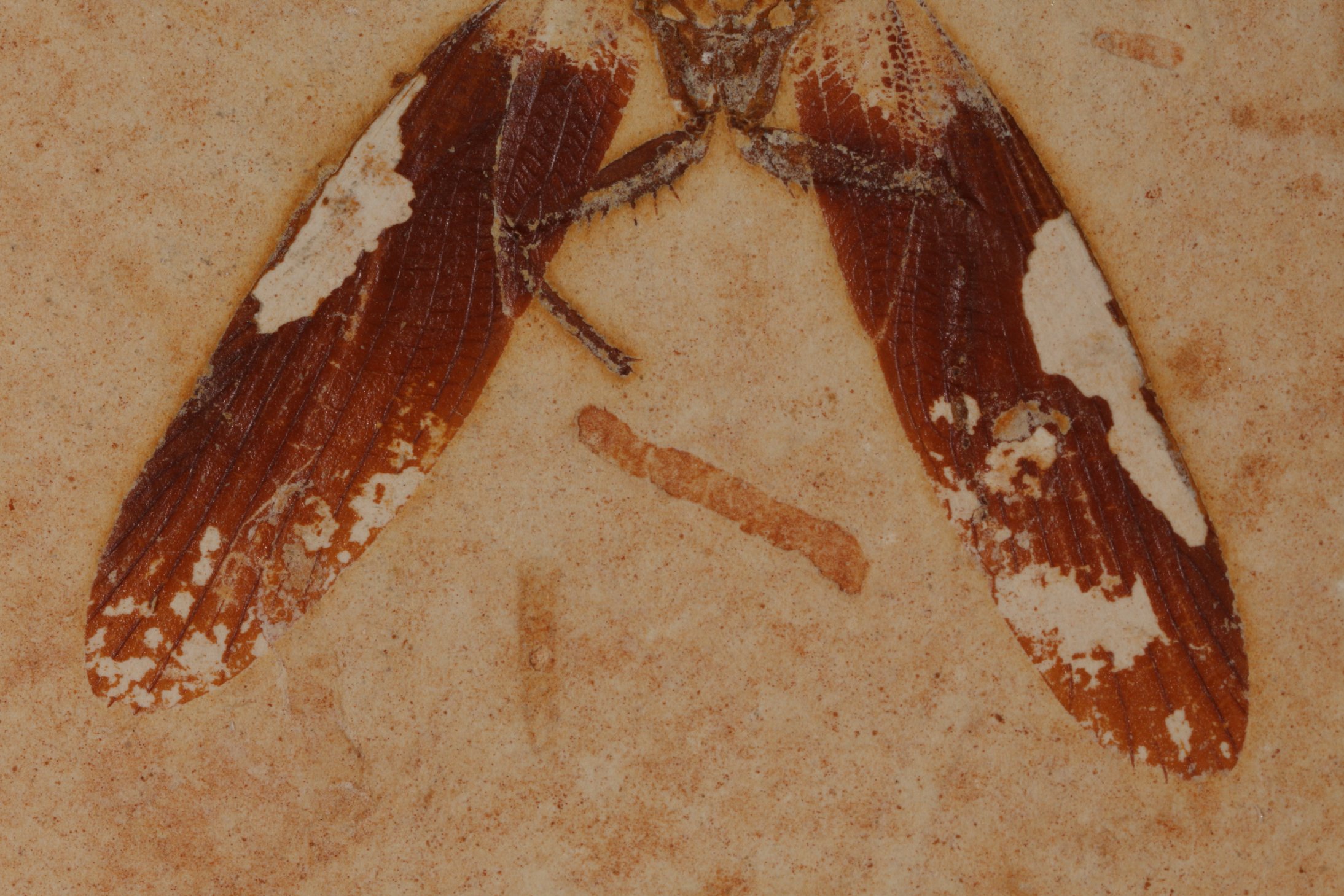

Supplement: Supplemental Information 1 [file peerj-05-3605-s001.zip › Santanmantis2_rawdata0009.jpg]

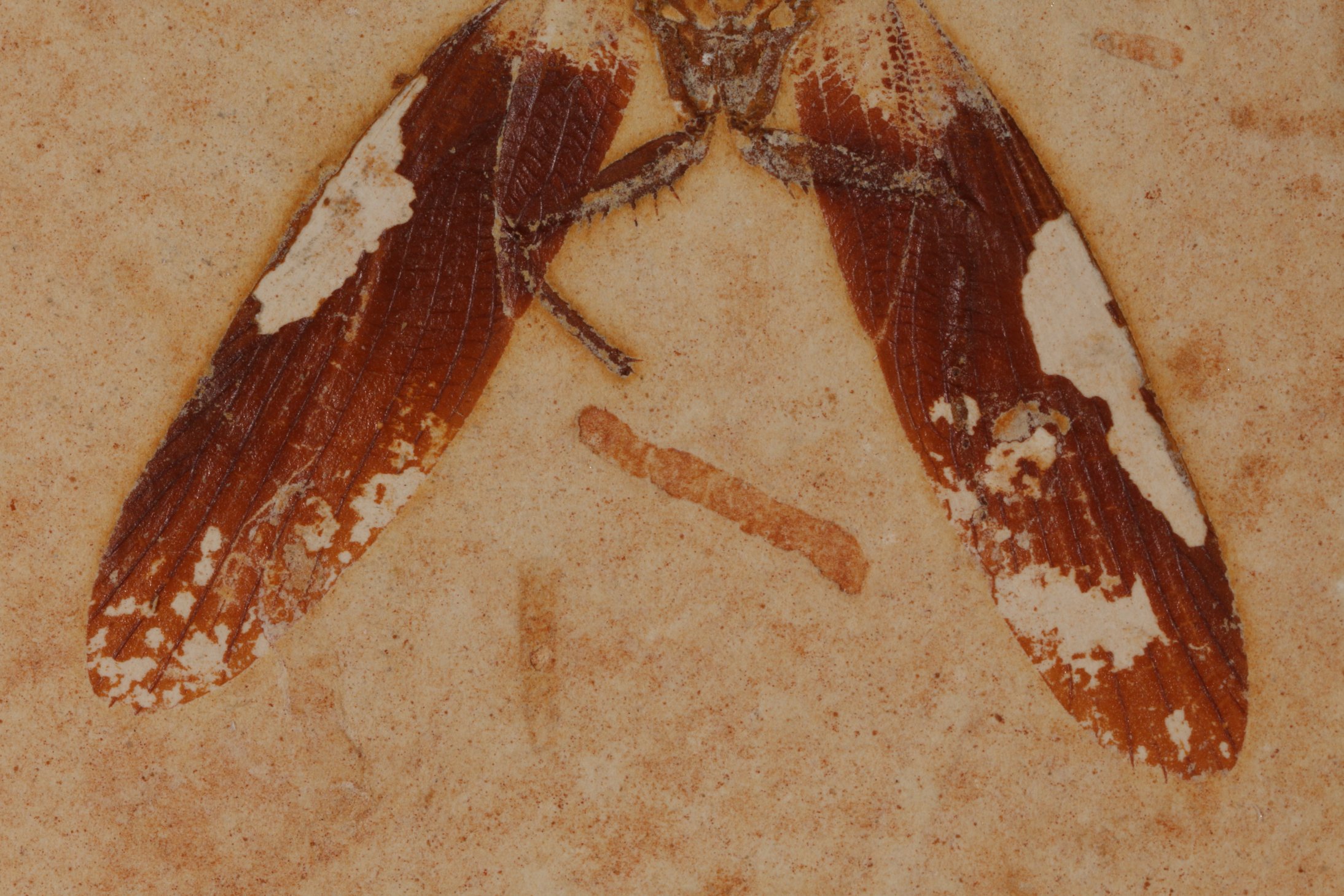

Supplement: Supplemental Information 1 [file peerj-05-3605-s001.zip › Santanmantis2_rawdata0010.jpg]

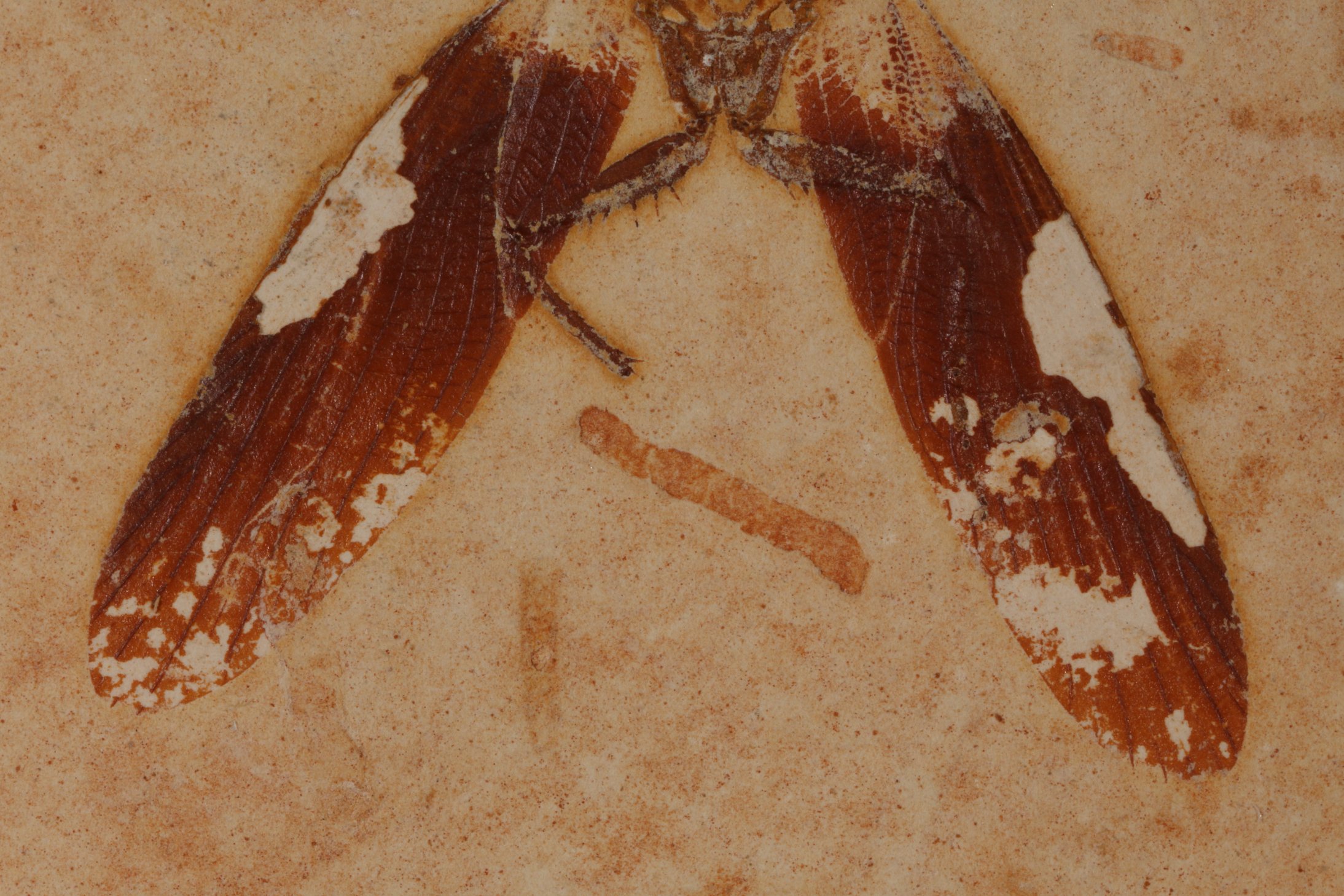

Supplement: Supplemental Information 1 [file peerj-05-3605-s001.zip › Santanmantis2_rawdata0011.jpg]

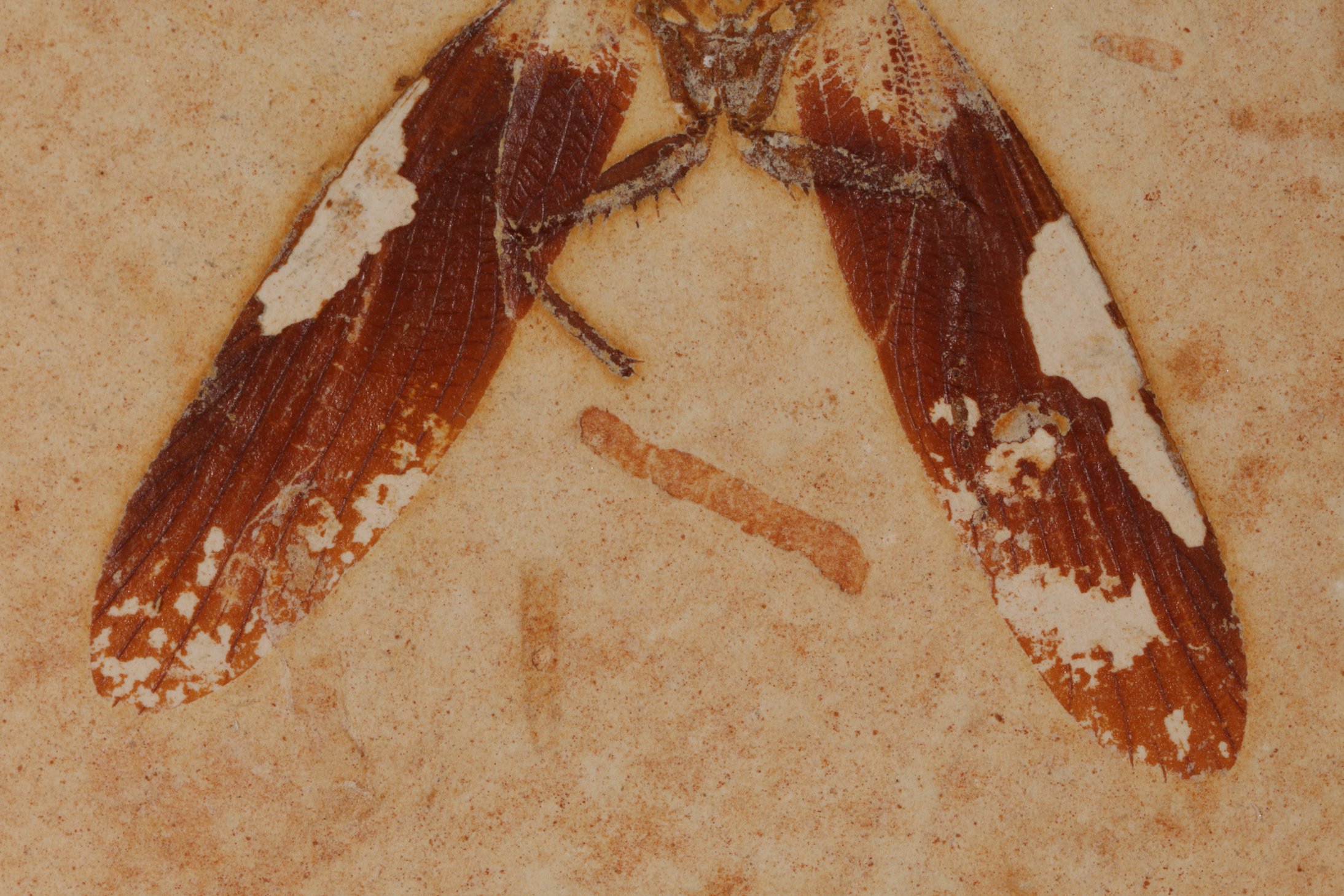

Supplement: Supplemental Information 1 [file peerj-05-3605-s001.zip › Santanmantis2_rawdata0012.jpg]

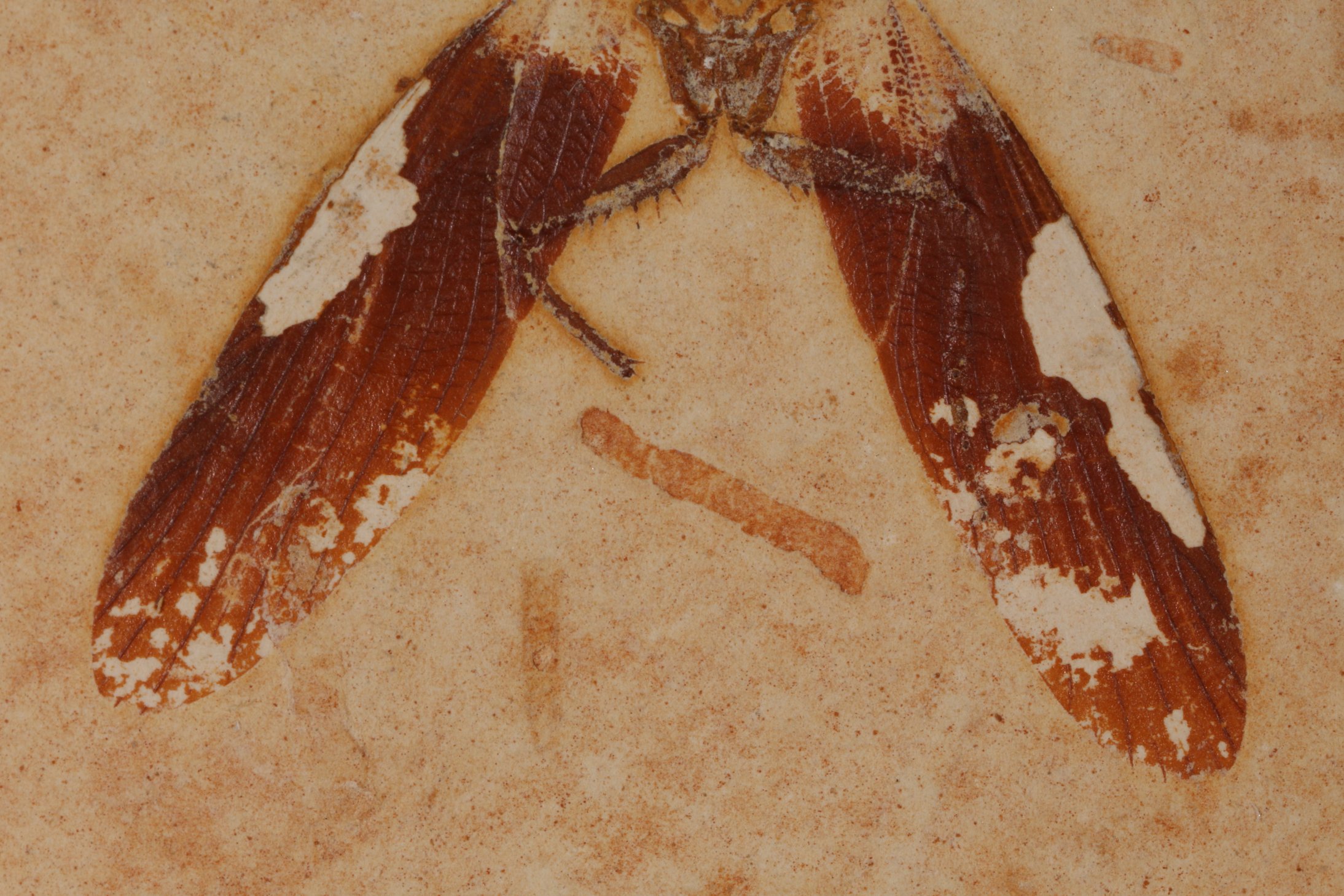

Supplement: Supplemental Information 1 [file peerj-05-3605-s001.zip › Santanmantis2_rawdata0013.jpg]

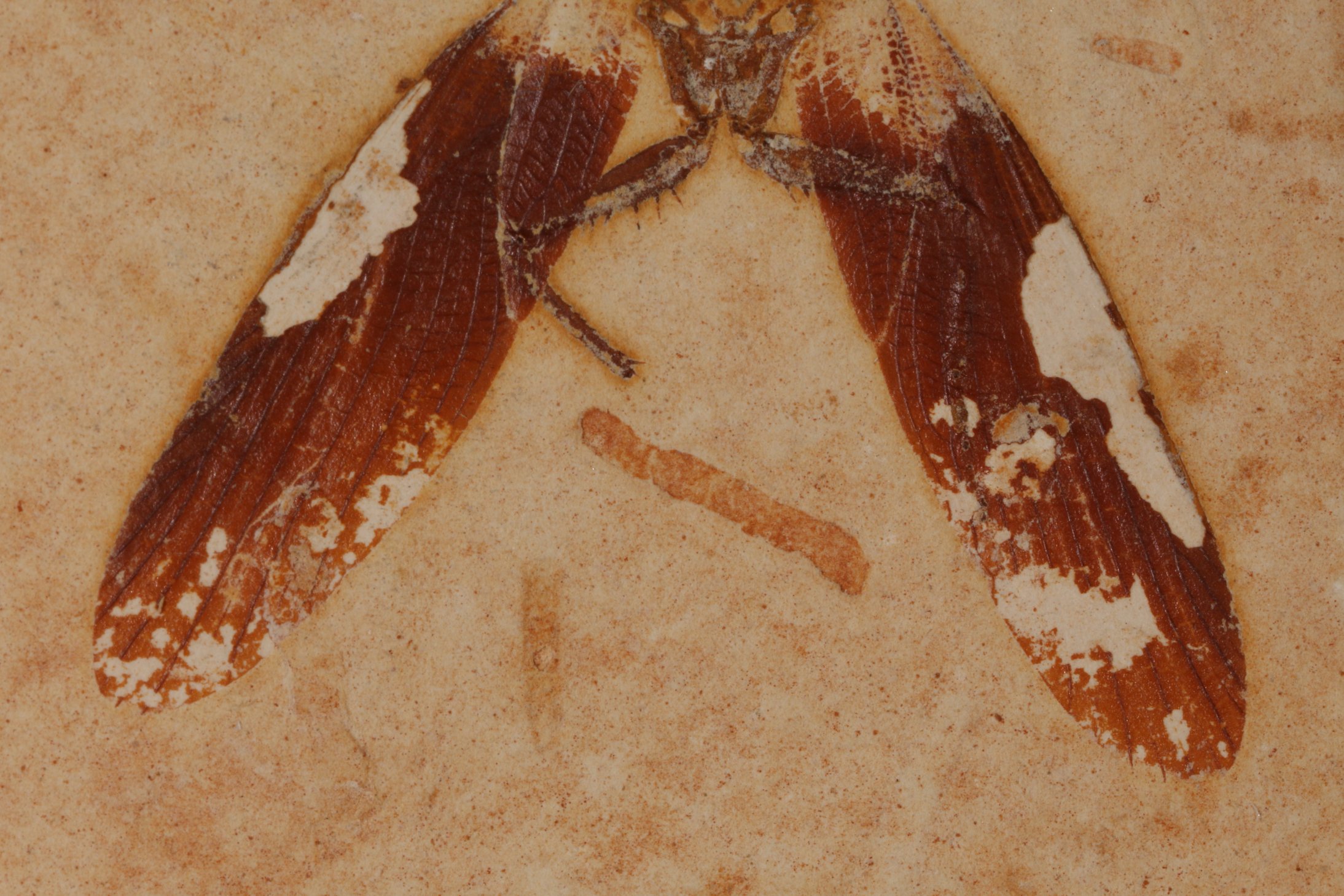

Supplement: Supplemental Information 1 [file peerj-05-3605-s001.zip › Santanmantis2_rawdata0014.jpg]

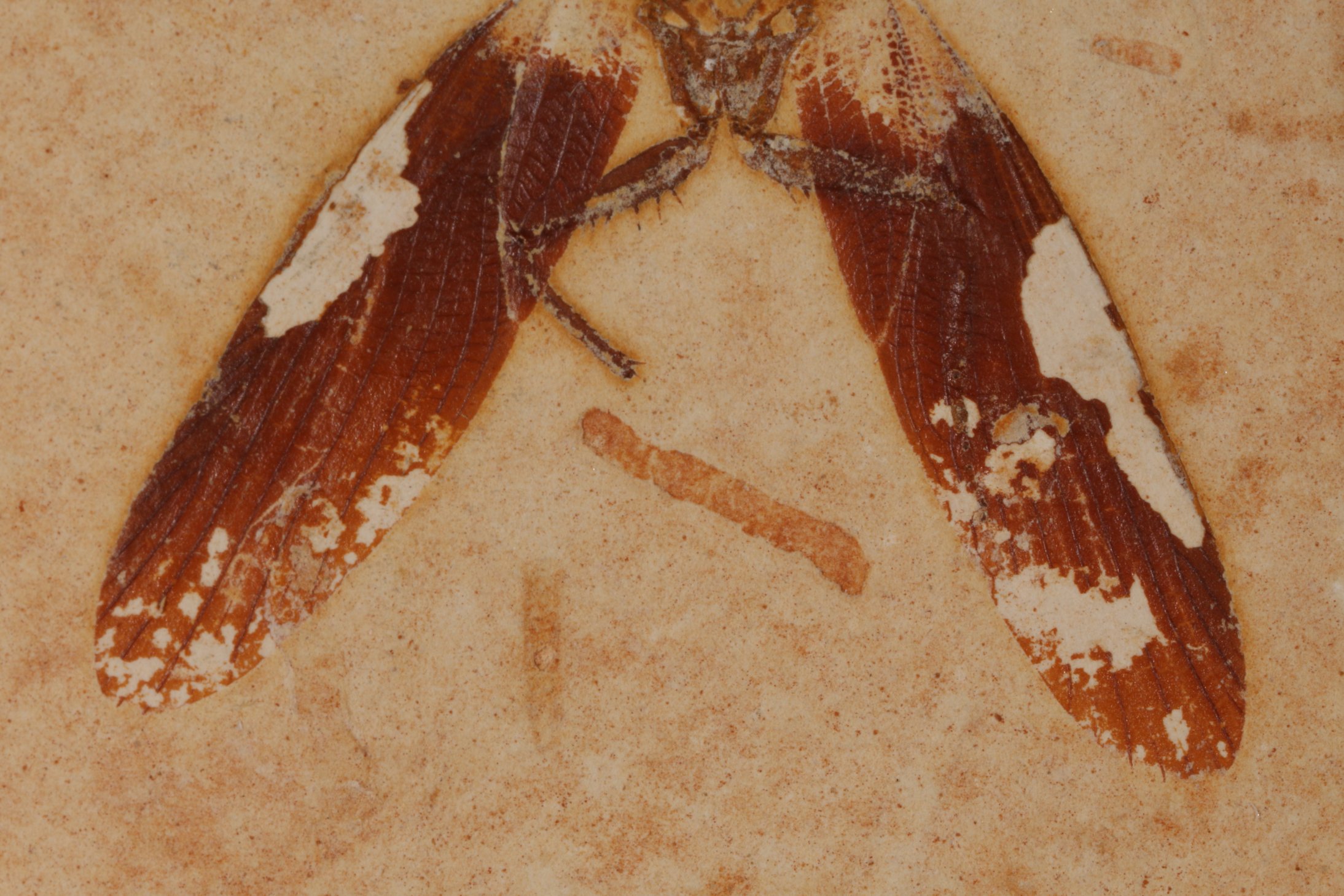

Supplement: Supplemental Information 1 [file peerj-05-3605-s001.zip › Santanmantis2_rawdata0015.jpg]

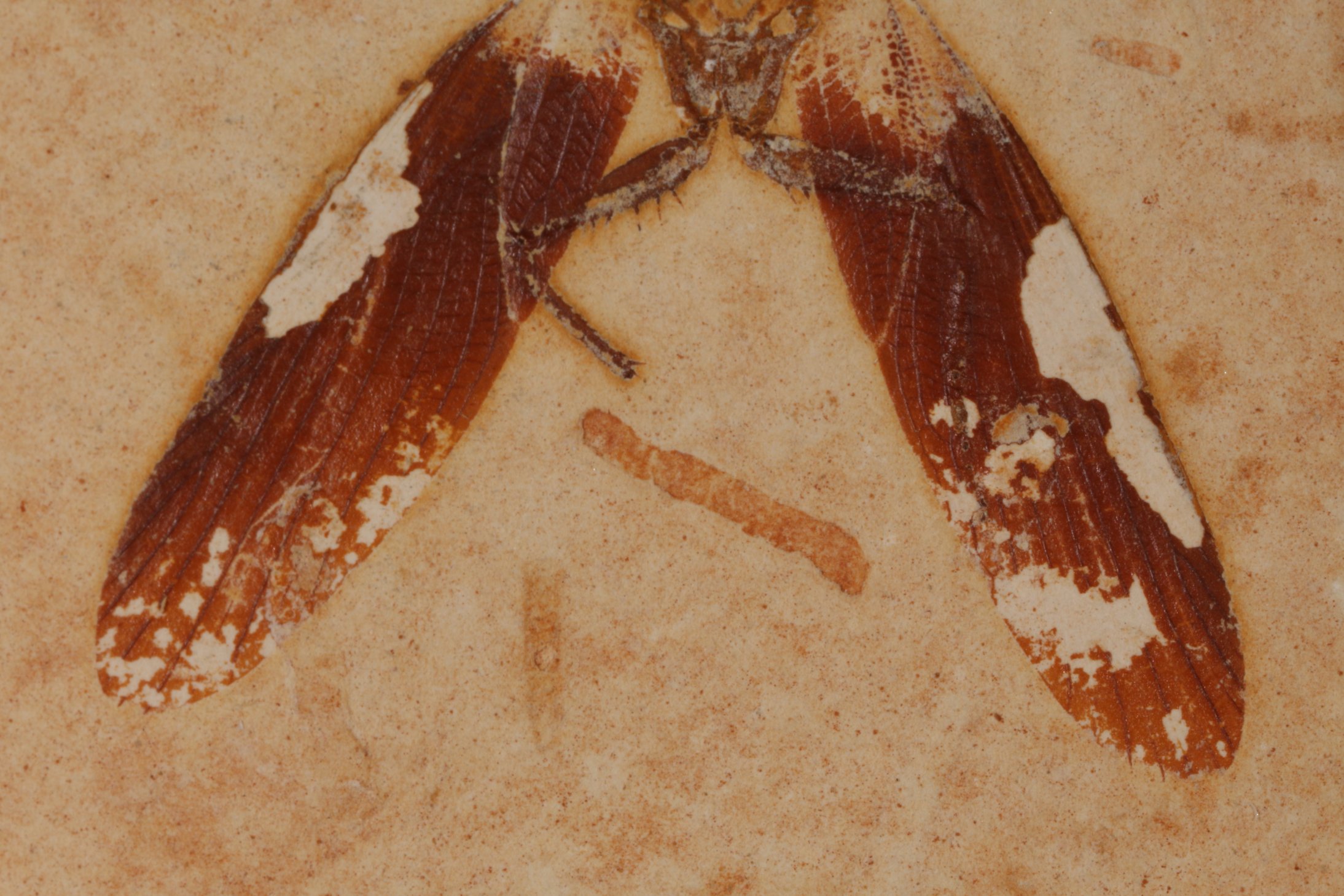

Supplement: Supplemental Information 1 [file peerj-05-3605-s001.zip › Santanmantis2_rawdata0016.jpg]

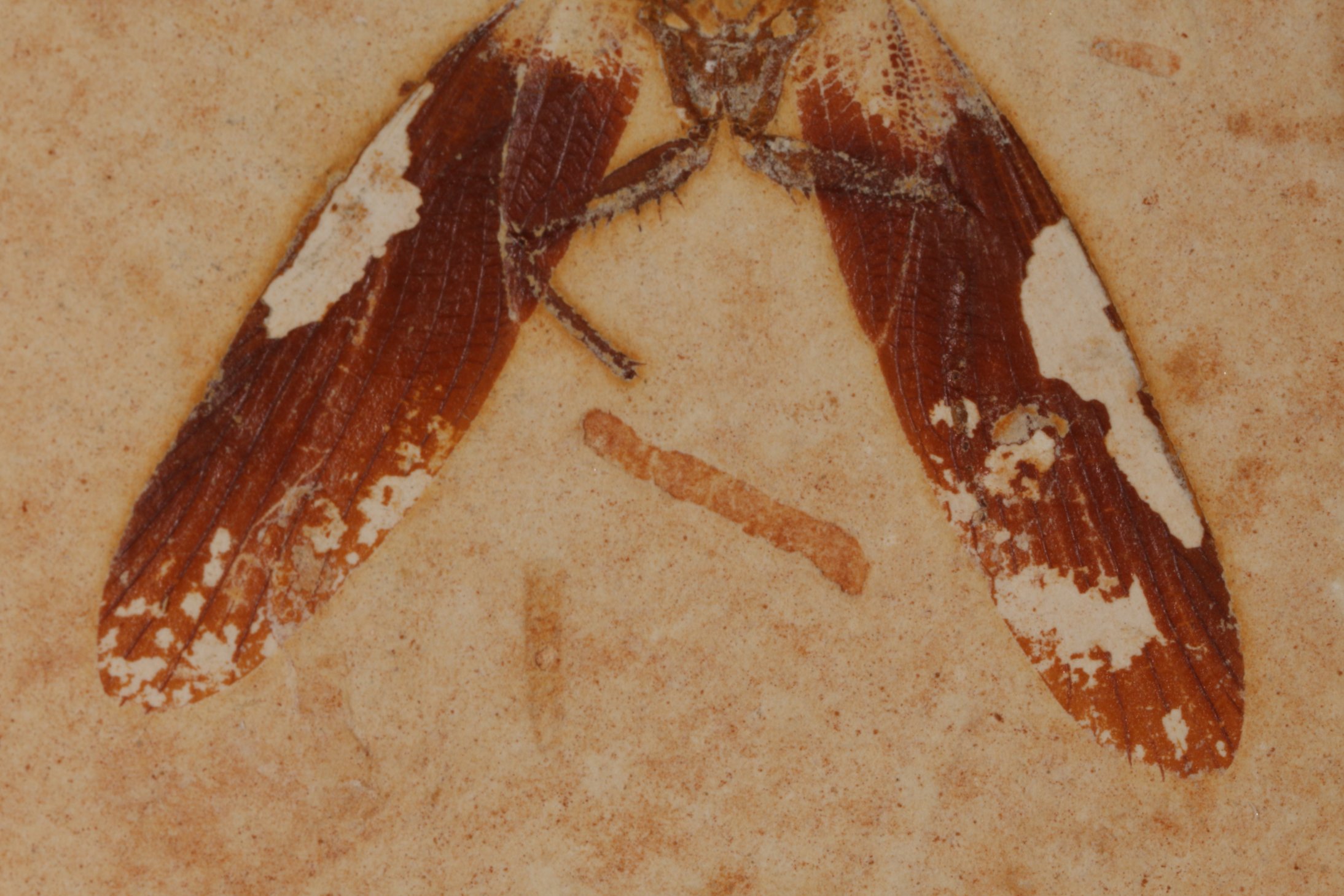

Supplement: Supplemental Information 1 [file peerj-05-3605-s001.zip › Santanmantis2_rawdata0017.jpg]

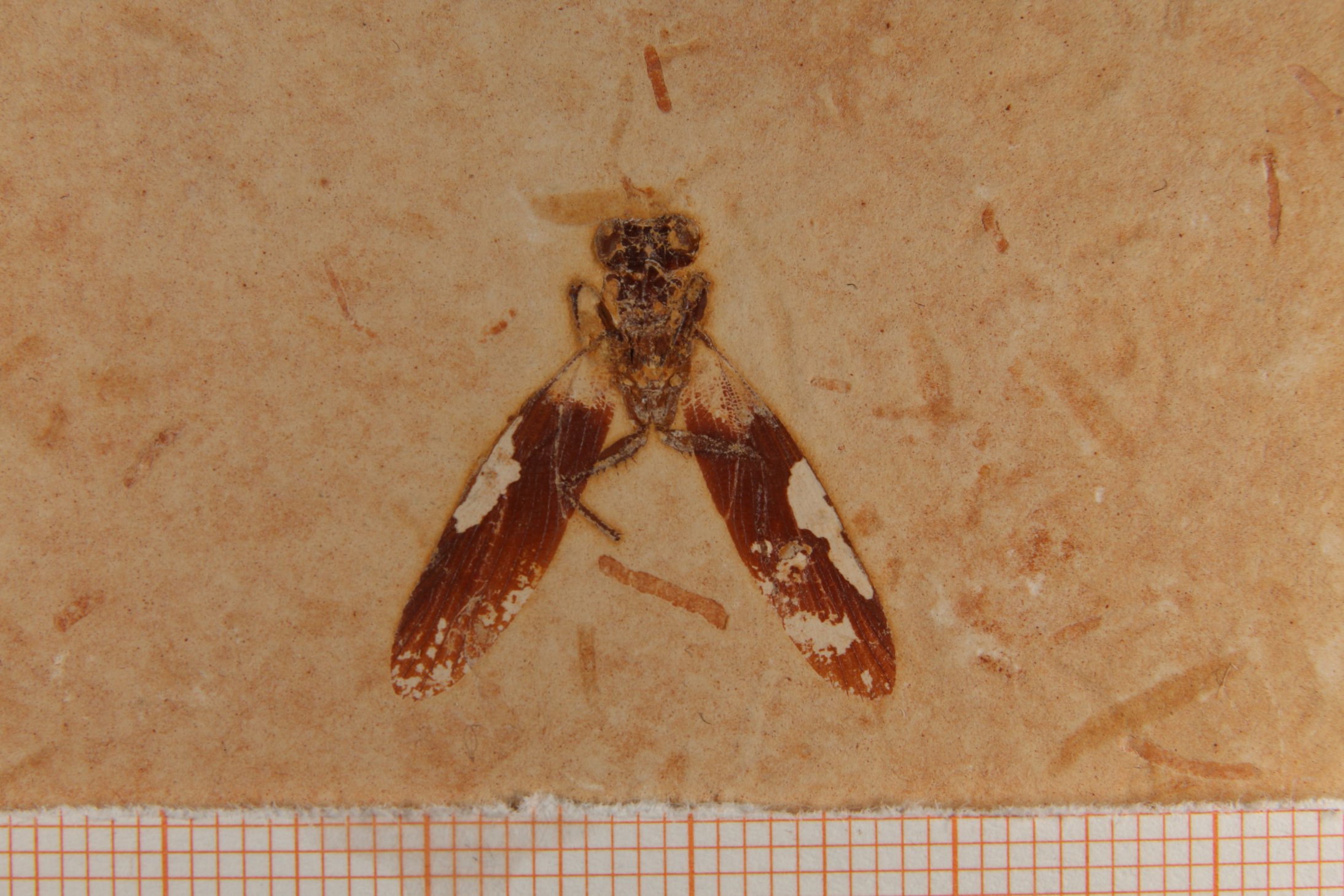

Supplement: Supplemental Information 1 [file peerj-05-3605-s001.zip › Santanmantis2_rawdata0018.jpg]

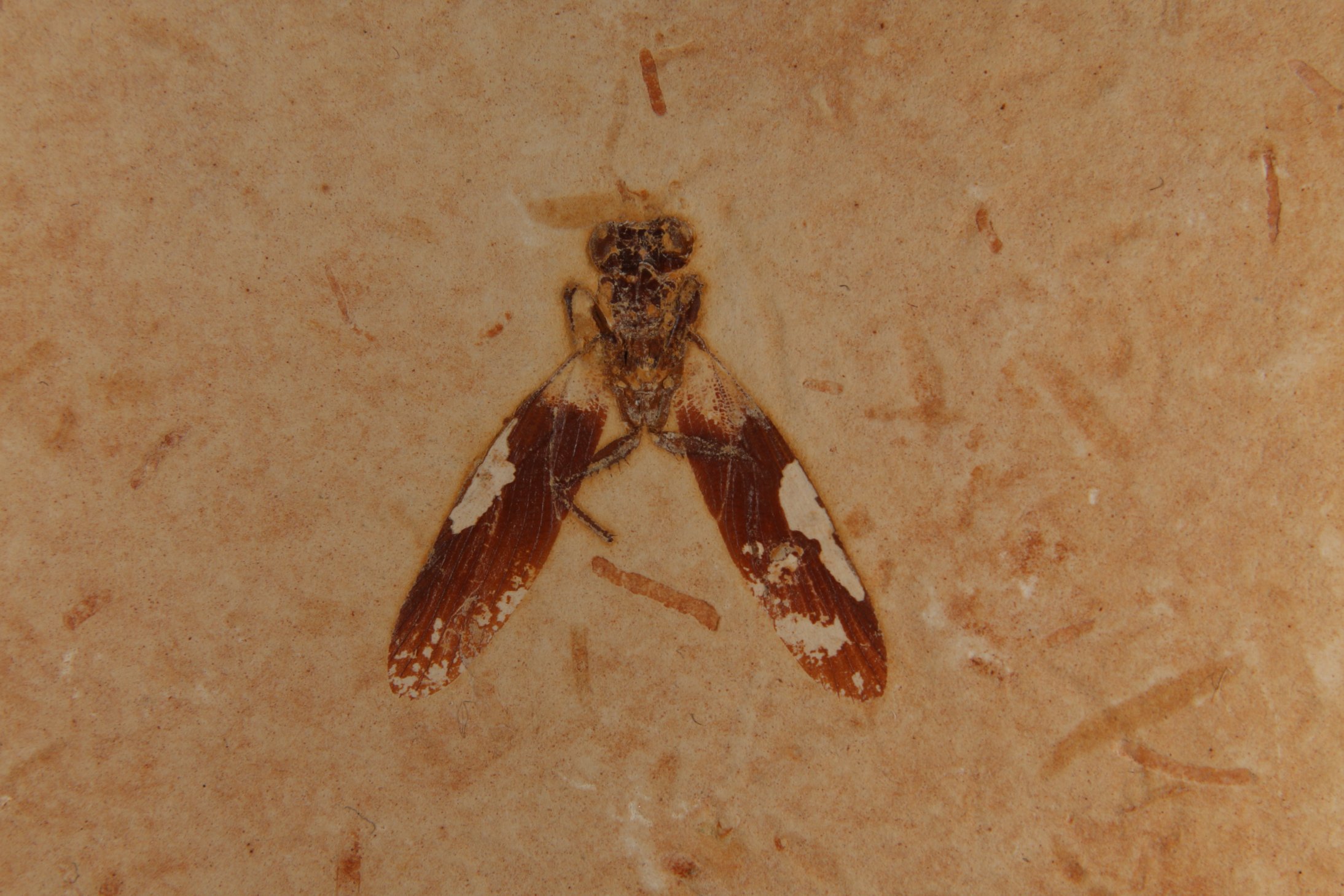

Supplement: Supplemental Information 1 [file peerj-05-3605-s001.zip › Santanmantis2_rawdata0019.jpg]

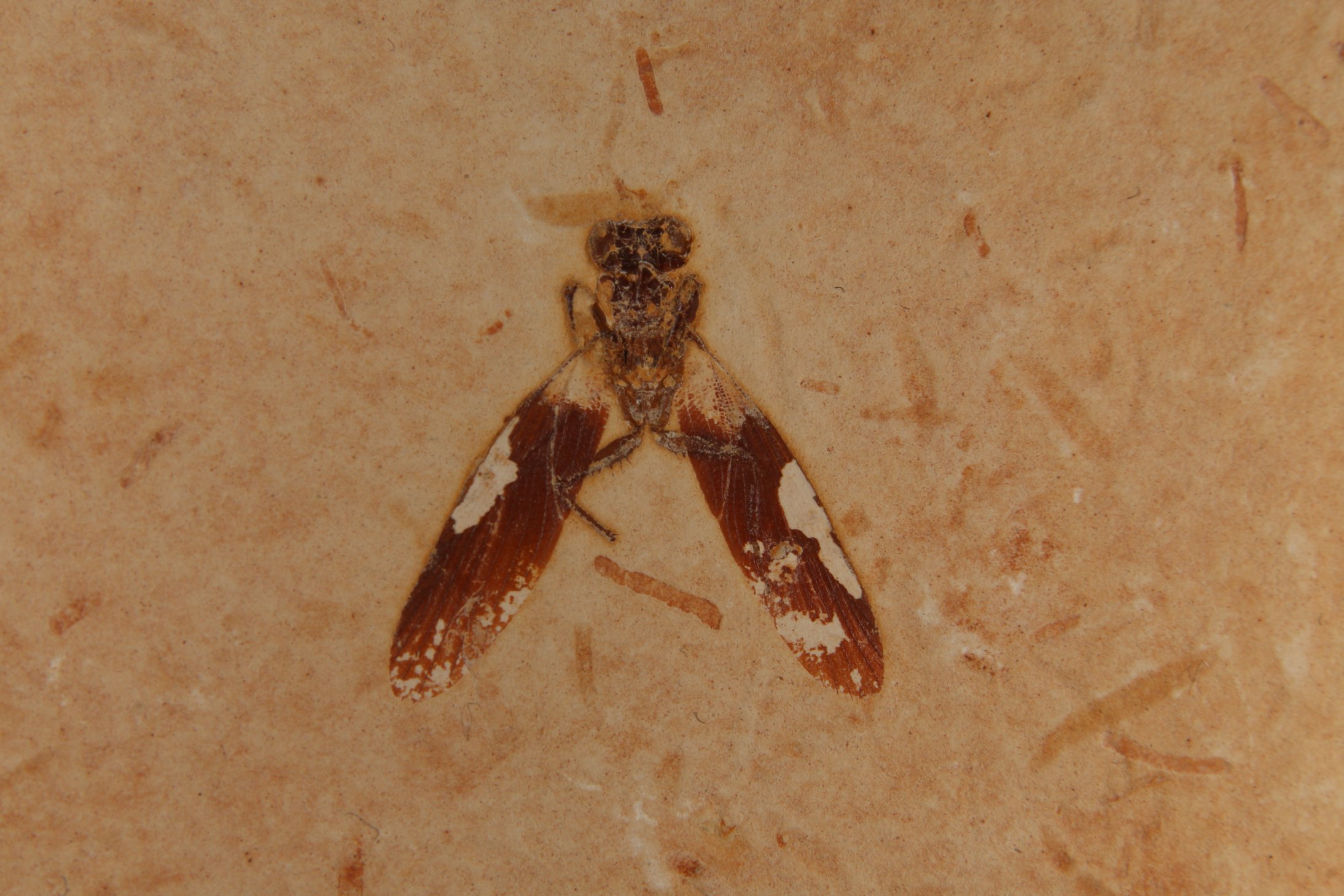

Supplement: Supplemental Information 1 [file peerj-05-3605-s001.zip › Santanmantis2_rawdata0020.jpg]
